# Dynamic Coordination Microenvironments of Fe Single-Atom Catalysts in Advanced Oxidation Processes: From Working-State Evolution to Pathway-Selective Regulation

**DOI:** 10.3390/molecules31193491

**Published:** 2026-10-01

**Authors:** Shuqi Li, Shun Li, Haobin Zheng, Xinyi Lou

**Affiliations:** 1 Institute of Ecology & Health, Hangzhou Polytechnic University, Hangzhou 311300, China; smoothlee2013@zju.edu.cn (S.L.);; 2 School of Environment and Spatial Informatics, China University of Mining and Technology, Xuzhou 221006, China

**Keywords:** iron single-atom catalysts, dynamic coordination, advanced oxidation processes, high-valent iron-oxo, interfacial electron transfer, catalyst durability

## Abstract

Iron single-atom catalysts (Fe-SACs) offer an atomically defined platform for peroxide-based advanced oxidation, yet their reactivity is still frequently rationalized using coordination structures measured only before or after catalysis. Such static descriptors identify precursor sites but do not necessarily resolve the Fe configurations that carry turnover. This Review establishes a dynamic coordination framework that distinguishes resting structures, reaction-induced working states, and irreversible deactivation products. It examines how first-shell coordination, axial ligation, extended microenvironments, neighboring sites, and interfacial solvation govern the formation and interconversion of peroxide-bound, hydroxy, Fe-oxo, and electronically coupled Fe-oxidant states. Radical, high-valent Fe-oxo, singlet-oxygen, and interfacial electron-transfer pathways are treated as competing catalytic fluxes emerging from a shared working-state landscape rather than as fixed attributes of individual Fe-N_x_ motifs. Particular emphasis is placed on the evidence required to identify kinetically competent intermediates, distinguish reversible coordination adaptation from structural escape, and connect working-state populations with regeneration and lifetime. Coordination and microenvironment engineering strategies are then evaluated according to their ability to control pathway selectivity without compromising site recoverability. Covalent organic frameworks are discussed as molecularly programmable platforms that can integrate Fe-site definition, charge transport, solvation, and oxidant supply. The resulting structure–state–pathway–lifetime framework advances Fe-SAC design from static activity correlations toward predictive control of catalytic trajectories under realistic operating conditions.

## 1. Introduction

### 1.1. Advanced Oxidation Processes, from Reactive Species to Dynamic Catalytic Interfaces

Advanced oxidation processes (AOPs) were originally developed around the generation of highly reactive oxidants, particularly hydroxyl radicals, for the destruction of contaminants resistant to conventional treatment [1,2,3,4]. This framework has expanded from classical Fenton and UV/H_2_O_2_ chemistry to photocatalytic, electrochemical, and persulfate-based processes [2,5]. Recent studies of single-atom catalysis and nonradical interfacial oxidation further show that atomic-scale coordination and reaction environment affect pathway selection [6,7]. The oxide-superstructure study [6] establishes a broader AOP principle, rather than direct evidence for an Fe single-atom working state. Oxidant decomposition or nominal reactive-species yield alone does not explain differences in oxidant utilization, pathway selectivity, mineralization, and environmental robustness [4,8]. The central mechanistic question has therefore shifted from how much reactive oxidant is generated to how the catalytic interface directs oxidizing equivalents into specific reaction pathways.

The conventional distinction between radical and nonradical AOPs remains useful for classification but is insufficient for mechanistic prediction. Freely diffusing ^•^OH and SO_4_^•−^ provide rapid and broadly reactive oxidation but are readily intercepted by dissolved organic matter and inorganic solutes [9,10,11]. PMS and PDS can also access singlet-oxygen, high-valent-metal, and surface-mediated electron-transfer pathways whose selectivity depends more strongly on interfacial chemistry [2,4,8]. These pathways are not intrinsic attributes of the oxidant. They emerge from competing elementary steps after oxidant coordination to the catalyst [12]. Molecular approach, desolvation, adsorption geometry, electron and proton transfer, substrate coadsorption, and interfacial water organization all precede or accompany O-O cleavage [12,13,14]. The operative catalytic entity is therefore better described as a catalyst–oxidant–pollutant–water ensemble than as an isolated active site reacting with a bulk oxidant [12].

Recent operando observations have made this distinction experimentally testable and mechanistically consequential. Oxidant adsorption can distort M-N_4_-C coordination environments, while time-resolved operando spectroscopy has directly resolved the formation and decay of surface Fe(IV)=O from an Fe-N_4_ precursor during PMS activation, thereby generating transient structures that cannot be inferred from precursor characterization alone [15,16]. Thus, the structure characterized before reaction may define the starting point of catalysis without representing the configuration that actually performs chemical turnover. This motivates a more fundamental description of heterogeneous AOPs based on how catalytic sites evolve upon oxidant binding, how those working states partition reaction flux, and whether they return reversibly after substrate conversion.

### 1.2. Fe Single-Atom Catalysts, Beyond Static Site Assignment Toward Dynamic Working States

Fe single-atom catalysts provide a particularly instructive platform for examining this problem, because their atomic dispersion enables direct interrogation of metal–environment relationships while exposing the limitations of static structural descriptions. Atomic dispersion minimizes the structural averaging inherent to nanoparticles and enables Fe coordination number, ligand identity, neighboring heteroatoms, and support structure to be treated as chemically meaningful variables [17,18,19,20]. Iron is additionally suited to peroxide activation because of its accessible valence and spin states and its ability to form electronically tunable coordination environments with N-, O-, S-, and C-containing supports [3,8,21,22,23,24,25].

Fe-N_4_ has consequently become the principal structural reference for Fe single-atom catalysis. X-ray absorption and Mössbauer spectroscopy established the importance of atomically dispersed Fe-Nx environments and provided a structural vocabulary for connecting Fe valence, spin, ligand field, and catalytic behavior [21,26,27,28]. However, Fe-N_4_ is a first-shell descriptor rather than a complete active-site identity. The same Fe-N_4_ notation can therefore correspond to electronically distinct states with different catalytic trajectories. Pyrolyzed Fe-N-C materials may contain electronically distinct Fe populations, heterogeneous N environments, carbon defects, and minority clusters that remain difficult to resolve by ensemble-averaged measurements [26,28,29]. Moreover, coordination features outside the first shell can substantially modify the behavior of an apparently unchanged Fe-N_4_ center.

The limitations of static assignment become more pronounced under reaction conditions. Changes in first-shell coordination can shift peroxide activation from one working-state manifold to another, whereas higher-shell heteroatoms and neighboring sites can regulate Fe-oxo formation, electronic polarization, and structural stability without changing the nominal Fe-N_4_ label [12,16,25,30,31,32,33,34]. Substrate electronics can further alter the relative contributions of radical and electron-transfer pathways even when catalyst structure and oxidant identity remain unchanged. The catalytic mechanism is therefore not uniquely encoded in the precursor coordination formula. It emerges from the dynamic population and kinetic connectivity of states accessible under reaction conditions.

This distinction cannot be resolved by static characterization alone. Atomic-resolution microscopy can establish spatial isolation, and EXAFS can constrain the average Fe coordination environment, but neither technique proves that the same structure persists during turnover [26]. Fe sites may reversibly acquire axial ligands, change valence or spin, distort out of plane, or access peroxide-, hydroxo-, and oxo-bound configurations. They may also undergo irreversible demetallation, migration, or aggregation [26,28]. A central challenge is therefore to distinguish reversible coordination dynamics that constitute catalysis from irreversible structural evolution that constitutes deactivation [28,29]. Operando and time-resolved measurements are required to connect the precursor site with the working-state population, catalytic flux, and regeneration kinetics.

### 1.3. Dynamic Coordination Microenvironments, Concept and Scope

We define the dynamic coordination microenvironment as the Fe-centered and experimentally traceable chemical environment that determines accessible catalytic states and evolves during turnover. It comprises not only first-shell ligands but also second- and higher-shell heteroatoms, axial ligands, neighboring atomic or molecular sites, support distortions, local solvation, proton networks, oxidants, intermediates, and adsorbed substrates when these components measurably alter Fe electronic structure, geometry, ligand field, adsorption, or regeneration [32,34,35]. This definition extends coordination chemistry beyond a static Fe-Nx formula while retaining a physically testable boundary between specific metal–environment interactions and nonspecific support effects.

Three structural regimes are distinguished throughout this Review. The resting state denotes the dominant Fe configuration before substantial catalytic turnover; the working state comprises reaction-induced peroxide-bound, hydroxo, oxo, electronically reorganized, or geometrically distorted configurations that participate directly in elementary steps; and the deactivated state comprises structures irreversibly removed from the productive cycle through Fe-ligand rupture, demetallation, migration, aggregation, or support degradation [26,31,35]. Only reversible structural evolution coupled to turnover is treated here as productive dynamic coordination. This distinction avoids the increasingly common tendency to describe any reaction-induced structural change as “productive dynamic coordination,” irrespective of whether it contributes to catalysis or reflects catalyst failure.

Within this framework, coordination structure governs catalysis across multiple, interconnected length scales. The first coordination shell determines Fe anchoring and local ligand-field symmetry. Higher-shell interactions and neighboring sites tune charge distribution and spin state, while axial ligation regulates access to oxidant-binding and bond-activation geometries. Pore architecture, interfacial water, proton activity, and substrate organization further control the population, lifetime, and consumption of catalytically relevant working states. Accordingly, the structure–activity relationship is best understood as a dynamic catalytic sequence linking the resting coordination state with oxidant binding, formation of reactive working states, substrate conversion, and eventual site regeneration or deactivation.

Covalent organic frameworks (COFs) are considered here as programmable platforms for interrogating dynamic coordination microenvironments, rather than as a separate materials theme. Their reticular structures permit deliberate changes to metal-binding pockets, linker electronics, pore dimensions, and solvation environments that are more difficult to isolate in pyrolyzed carbons [36,37,38]. The rationale for including COFs is their potential to test whether encoded structural variables control the population and regeneration of Fe working states. Evidence from Fe-containing frameworks supports structural and electronic tunability, whereas observations in Co frameworks and peroxide-producing COFs provide mechanistic analogies only [36,39,40,41,42,43,44,45]. Direct operando observation of a complete Fe-COF working-state cycle during pollutant oxidation remains limited.

Accordingly, this Review is organized around the evolution of the Fe catalytic center rather than a catalogue of catalyst compositions. We first establish resting and reaction-induced coordination states and the evidence required to assign them, then connect those states to pathway branching and regeneration, and finally examine how first-shell, extended, cooperative, and framework-defined microenvironments can be engineered to control reversible Fe-state evolution. The overarching objective is to move Fe single-atom Fenton chemistry from static structure–activity correlations toward a dynamic structure–state–pathway–lifetime framework.

Mechanistic claims in this Review are evaluated using a four-tier evidence framework. Direct evidence requires time-resolved structural or spectroscopic observation of an Fe working state under relevant reaction conditions. Convergent support requires mutually consistent kinetic, isotopic, probe-based, and computational results. Mechanistic inference denotes a chemically plausible state that has not been observed directly. A design proposition is a testable hypothesis extrapolated from established coordination principles. Results from oxygen reduction, ozonation, peracetic-acid activation, photocatalytic H_2_O_2_ synthesis, and non-Fe single-atom systems are identified as cross-reaction analogies; they do not independently establish an Fe-SAC advanced-oxidation mechanism.

## 2. From Resting Coordination Structures to Reaction-Induced Fe Working States

### 2.1. Resting-State Coordination Motifs

Resting Fe sites are most commonly described by first-shell coordination number and ligand identity. However, these descriptors should be regarded as structural constraints defining the initial coordination ensemble rather than complete representations of the catalytic site. Fe-N_4_ serves as the principal reference, while Fe-N_3_ and Fe-N_2_ represent progressively less constrained coordination motifs rather than intrinsically more active structures. These labels are useful because they describe the initial ligand field and available coordination space. They should not, however, be interpreted as molecularly unique structures or as direct mechanistic labels.

Pyrolyzed Fe-N-C materials commonly contain several Fe populations, chemically inequivalent N atoms, structural disorder, and minor Fe-containing phases. The experimentally observed resting state should therefore be understood as a distribution of Fe environments with different probabilities of entering specific catalytic trajectories. EXAFS coordination numbers are ensemble averages, and overlapping Fe-N, Fe-O, and Fe-C scattering paths complicate unique structural assignment. The absence of a detectable Fe-Fe path supports atomic dispersion but cannot specify the complete Fe coordination environment. The role of a resting-state formula is thus to define a constrained precursor ensemble from which catalytically competent states can emerge under turnover.

#### 2.1.1. Fe-N_4_ as a Structural Reference

**Figure 1:**
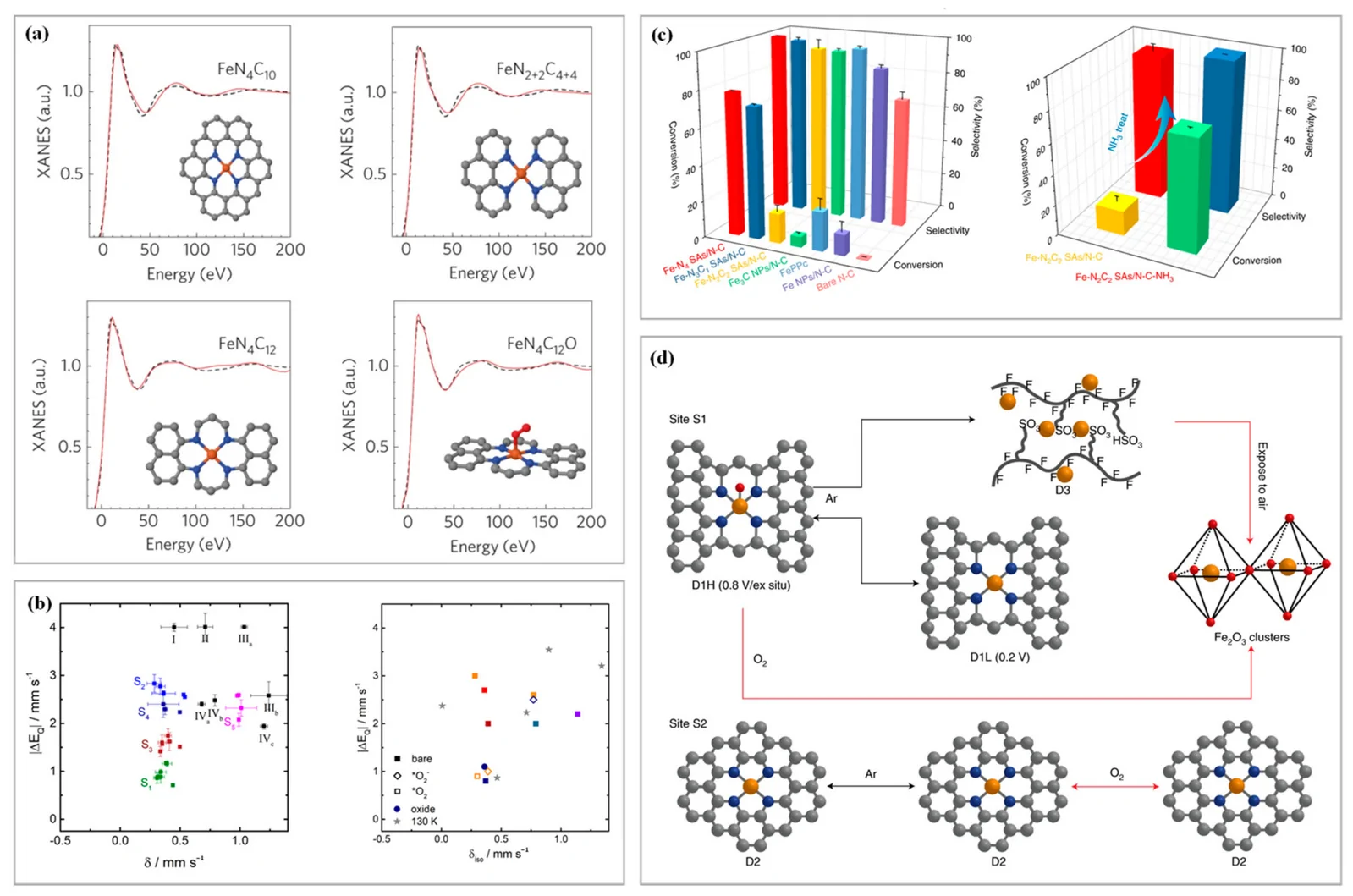
Fe-N_4_ as a Structural Reference. (**a**) Four equatorial N atoms anchor Fe while axial sites remain accessible. Reprinted with permission from Ref. [21]. Copyright 2015, Springer Nature. (**b**) Mössbauer-resolved Fe populations show that one first-shell label spans distinct states. Reprinted with permission from Ref. [27]. Copyright 2022, American Chemical Society. (**c**) Coordination number changes the accessibility of Fe-oxo chemistry. Reprinted with permission from Ref. [22]. Copyright 2019, Springer Nature. (**d**) Similar FeN_4_ assignments can follow different operational trajectories. Reprinted with permission from Ref. [28]. Copyright 2021, Springer Nature.

Fe-N_4_ became the canonical Fe single-atom motif because four equatorial N ligands provide strong metal anchoring while leaving axial directions available for dynamic coordination with solvent molecules, oxidants, and reaction intermediates. Combined X-ray absorption and ^57^Fe Mössbauer spectroscopy established porphyrin-like FeN_4_C_x_ motifs as important Fe-centered structures in pyrolyzed Fe-N-C materials (Figure 1a) [21]. Subsequent studies further showed that nominal Fe-N_4_ populations can differ in N type, carbon topology, spin state, axial coordination, and long-range electronic environment (Figure 1b–d) [22,24,27,28,35]. Thus, “Fe-N_4_” specifies first-shell composition rather than a unique electronic state. Pyrrolic and pyridinic coordination alter ligand-field splitting, spin-state distribution, and redox accessibility; nearby vacancies redistribute charge and strain; axial ligands change the (dz^2^) manifold; and remote heteroatoms modify the local electric field without changing the four primary Fe-N bonds. An Fe-N_4_ assignment is therefore most useful as a reference against which additional structural perturbations are measured.

This distinction is particularly important in Fenton-like chemistry. Fe-N_4_ precursors have been associated with ^1^O_2_ generation, Fe-oxo formation, direct electron transfer, and radical pathways under different reaction environments [23,33]. These divergent behaviors do not constitute contradictory assignments. Instead, they show that nominally identical first-shell motifs can evolve into different working states as oxidant binding and the surrounding microenvironment change.

Carbon topology provides a clear example. Vacancies adjacent to Fe-N_4_ can alter Fe electronic structure and facilitate the formation of oxygen-coordinated states while retaining atomic Fe dispersion [33]. Long-range defects can likewise influence neighboring Fe-N_4_ centers through the conjugated carbon lattice [30]. The catalytic information contained in the Fe-N_4_ notation therefore extends beyond the four Fe-N bonds. The first coordination shell is the primary structural anchor within a hierarchically coupled catalytic microenvironment.

#### 2.1.2. Coordinatively Unsaturated and Asymmetric Fe-N_x_ Sites

**Figure 2:**
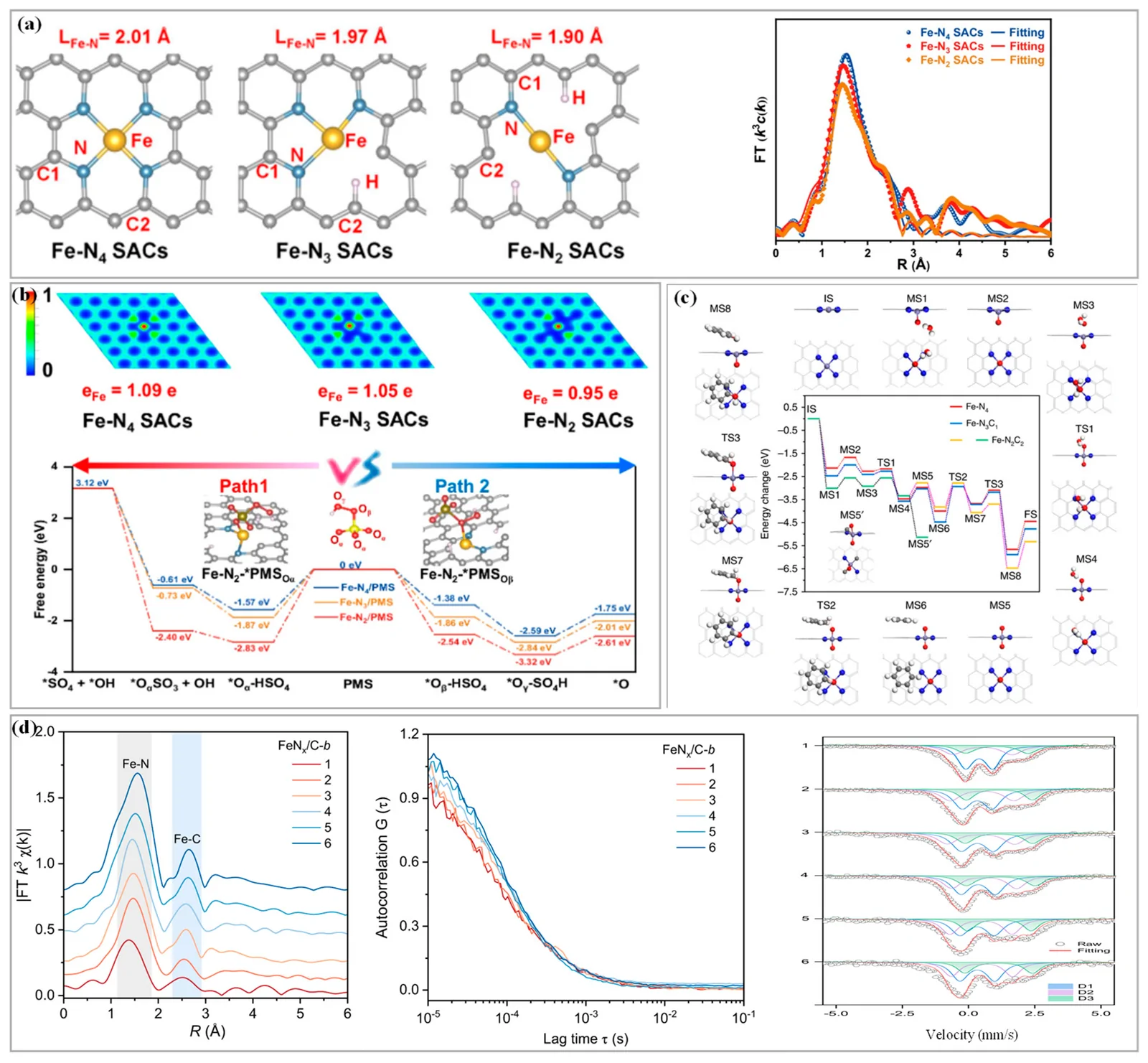
Reaction-dependent coordination–reactivity relationships in Fe single-atom catalysis. (**a**) Structural models and EXAFS validation of matched Fe-N_4_, Fe-N_3_, and Fe-N_2_ sites. Reprinted with permission from Ref. [25]. Copyright 2024, Springer Nature. (**b**) Coordination-dependent Fe charge and free-energy profiles for PMS activation on the matched Fe-Nx sites. The asterisk (*) denotes a surface site; species preceded by * are surface-bound intermediates rather than free radicals in solution. Reprinted with permission from Ref. [25]. Copyright 2024, Springer Nature. (**c**) Reaction-network energetics for H_2_O_2_-driven benzene oxidation over Fe-N_4_, Fe-N_3_C_1_, and Fe-N_2_C_2_ configurations. Reprinted with permission from Ref. [22]. Copyright 2019, Springer Nature. (**d**) Spectroscopic heterogeneity among nominal Fe-Nx populations, illustrating why ensemble-averaged coordination numbers do not uniquely identify the catalytically dominant site. Reprinted with permission from Ref. [46]. Copyright 2023, Springer Nature.

Reducing the number of N ligands changes both geometric accessibility and Fe electronic structure. However, the catalytic consequence of undercoordination depends on whether the additional coordination space is productively occupied during turnover. A matched Fe-N_2_/Fe-N_3_/Fe-N_4_ series provides one of the clearest demonstrations that coordination number regulates working-state accessibility (Figure 2a,b). In PMS activation, decreasing N coordination in this specific PMS activation system increased the formation of Fe(IV)=O, with Fe-N_2_ facilitating both oxidant activation and release of sulfate-derived intermediates [25]. Importantly, the trend does not establish a universal hierarchy of Fe-N_4_ < Fe-N_3_ < Fe-N_2_.

In H_2_O_2_-mediated benzene oxidation, a more highly coordinated Fe-N_4_ environment favored productive Fe-oxo chemistry relative to less N-coordinated analogues (Figure 2c) [22]. These contrasting examples demonstrate that coordination number acts as a pathway regulator rather than a universal activity descriptor. The relevant trade-off is between coordination accessibility and coordination stability. A less saturated Fe center may bind oxidant more readily but also stabilize adsorbates too strongly or undergo excessive out-of-plane displacement. Conversely, a more confined site may preserve structural integrity while imposing a higher barrier to formation of the required intermediate. The optimum coordination number consequently depends on oxidant identity, solvent, proton activity, substrate chemistry, and the rate-controlling step. Structural claims for Fe-N_2_ and Fe-N_3_ should also be treated cautiously (Figure 2d) [46]. An EXAFS-derived coordination number near three may represent a uniform Fe-N_3_ population, a mixture of Fe-N_2_ and Fe-N_4_, or a distorted Fe-N_4_ ensemble. Moreover, a low-coordinate site characterized in the dry state may immediately bind H_2_O or OH^−^ after immersion. The chemically relevant meaning of undercoordination is therefore the availability of coordination space, not necessarily persistence of a formally two- or three-coordinate Fe atom in water.

#### 2.1.3. Symmetry Breaking Beyond Coordination Number

**Figure 3:**
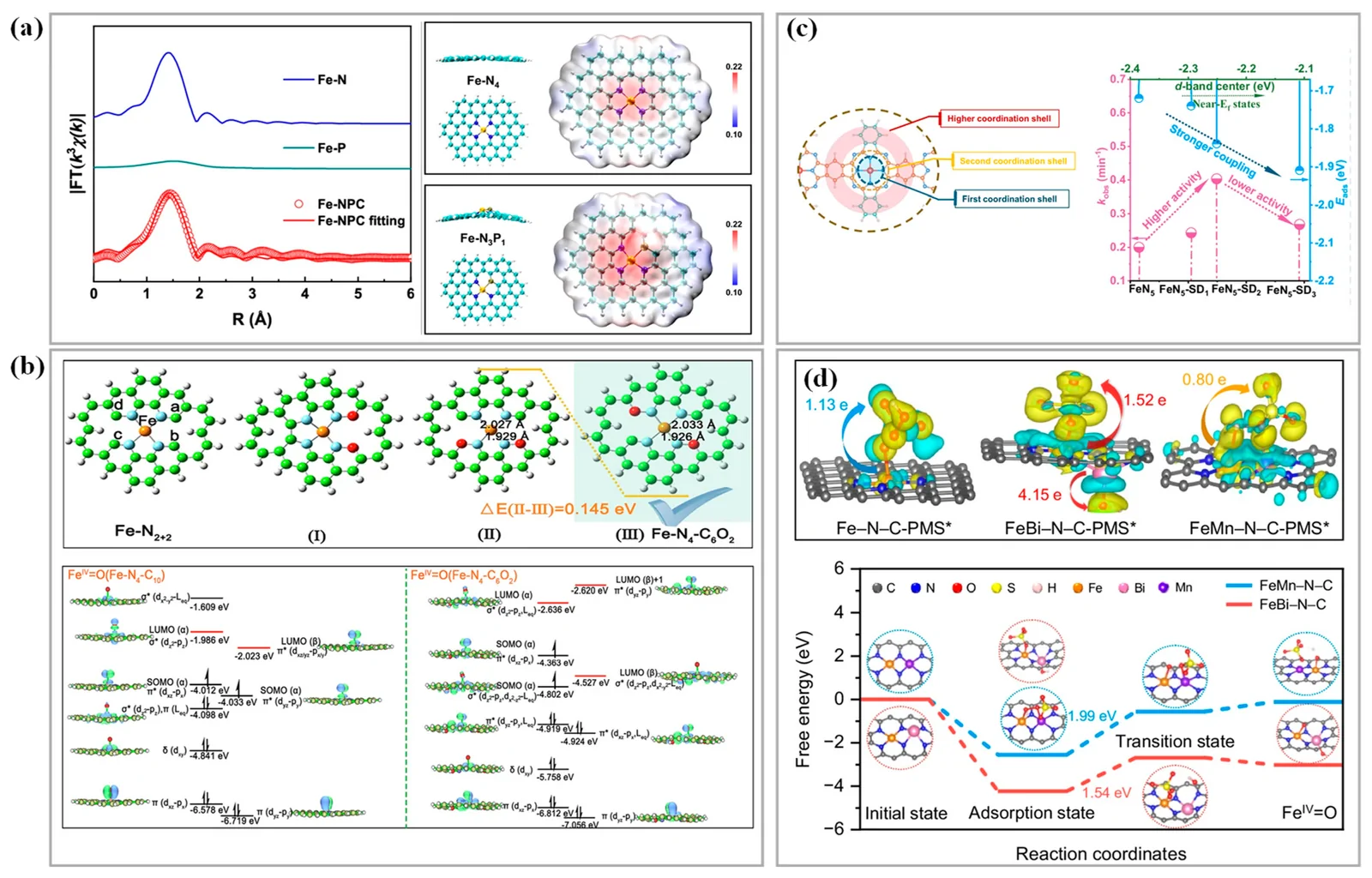
Multiscale symmetry breaking and coordination regulation of Fe single-atom sites beyond the nominal Fe-Nx descriptor. (**a**) First-shell symmetry breaking through P substitution in Fe-N_3_P_1_ sites. Reprinted with permission from Ref. [47]. Copyright 2025, Springer Nature. (**b**) Second-shell O coordination in Fe-N_4_-C_6_O_2_ modulates the electronic structure and Fe(IV)=O orbital energetics while preserving the first-shell Fe-N_4_ framework. Reprinted with permission from Ref. [16]. Copyright 2025, Springer Nature. (**c**) Higher-shell structural defects in FeN_5_-SD_x_ remotely regulate the electronic structure and PMS coupling of Fe-N_5_ sites, with FeN_5_-SD_2_ providing an optimized coordination environment for selective ^1^O_2_ generation. Reprinted with permission from Ref. [31]. Copyright 2025, Springer Nature. (**d**) Neighboring p-block-metal coordination in FeM_p_-N-C (M_p_ = Bi, In, and Sb), exemplified by FeBi-N-C, creates a cooperative PMS-binding microenvironment that enhances interfacial electron transfer and promotes selective Fe(IV)=O formation. The asterisk (*) denotes adsorbed PMS. Reprinted with permission from Ref. [34]. Copyright 2025, Springer Nature.

Coordination asymmetry can be introduced without simply removing an N ligand. First-shell heteroatom substitution, axial coordination, second-shell atoms, and remote defects all generate anisotropic electronic fields around Fe. Their common function is to alter charge distribution, ligand-field splitting, spin occupation, and the orientation by which an oxidant approaches the metal (Figure 3a) [47]. This concept expands the traditional coordination-number paradigm toward electronic and geometric microenvironment engineering.

First-shell substitution can exert a large electronic perturbation because the heteroatom participates directly in Fe bonding. However, structural assignments such as Fe-N_3_X or Fe-N_2_X_2_ frequently depend on model-sensitive EXAFS fitting when X is a light atom. For a critical structure–mechanism analysis, the important distinction is therefore not simply which heteroatom is present, but whether its position relative to Fe has been experimentally constrained.

Recent work illustrates why perturbations outside the first shell deserve equal attention. Second-shell oxygen in Fe-N_4_-C_6_O_2_ preserves the primary Fe-N_4_ framework while modifying the local electric field (Figure 3b) [16]. Higher-shell defects around well-defined Fe-N_5_ centers generate a nonmonotonic relationship among defect density, PMS adsorption, and catalytic response (Figure 3c) [31]. Neighboring p-block atoms can likewise redistribute charge around Fe and alter its spin-dependent response to PMS (Figure 3d) [34]. Axial oxygen coordination can switch the spin state of an Fe phthalocyanine center and open the 3dz^2^ orbital to peroxide interaction [48]. These observations establish a hierarchy of structural descriptors. First-shell coordination defines the primary ligand field, axial ligation regulates orbital accessibility, and higher-shell environments tune the electronic, electrostatic, and mechanical boundary conditions that govern working-state evolution. Resting-state assignment should therefore capture the minimum structural ensemble needed to explain working-state chemistry rather than seek a universally transferable Fe-Nx formula.

Collectively, resting-state coordination motifs should not be regarded as isolated molecular structures but as precursors that define the accessible catalytic landscape. First-shell coordination determines the fundamental ligand field, while axial ligands, second-shell atoms, defects, and neighboring sites reshape the probability of entering specific working states. This hierarchical coordination concept provides the structural basis for understanding why chemically similar Fe sites can diverge into distinct oxidation pathways under different reaction environments.

### 2.2. Oxidant-Regulated Working-State Evolution

Oxidant adsorption is itself a coordination event. When oxygen-containing oxidants enter the Fe coordination sphere, Fe-O interactions reshape the local coordination number, electronic population, and geometry before bond activation occurs. The oxidant-bound precursor should therefore be considered an early working state rather than a passive adsorbate on an unchanged resting site. This transition can involve solvent displacement, axial coordination, out-of-plane Fe displacement, charge redistribution, and local proton-network reorganization before O-O cleavage becomes kinetically accessible.

These working states are dynamic and often heterogeneous. The experimentally observed state should therefore be treated as a population distribution rather than a single molecular configuration. A nominal Fe-N_4_ precursor may populate Fe-N_4_-OOH, Fe-N_4_-OH, Fe-N_4_=O, or less well-defined Fe-oxidant complexes, whereas an undercoordinated site may become formally five- or six-coordinate after hydration and peroxide binding. The notation used below identifies chemically meaningful classes rather than a single frozen geometry. The mechanistic task is to determine which oxidant-bound states are populated, how rapidly they interconvert, and which states carry measurable catalytic flux.

#### 2.2.1. H_2_O_2_-Bound Fe-OOH Intermediates

**Figure 4:**
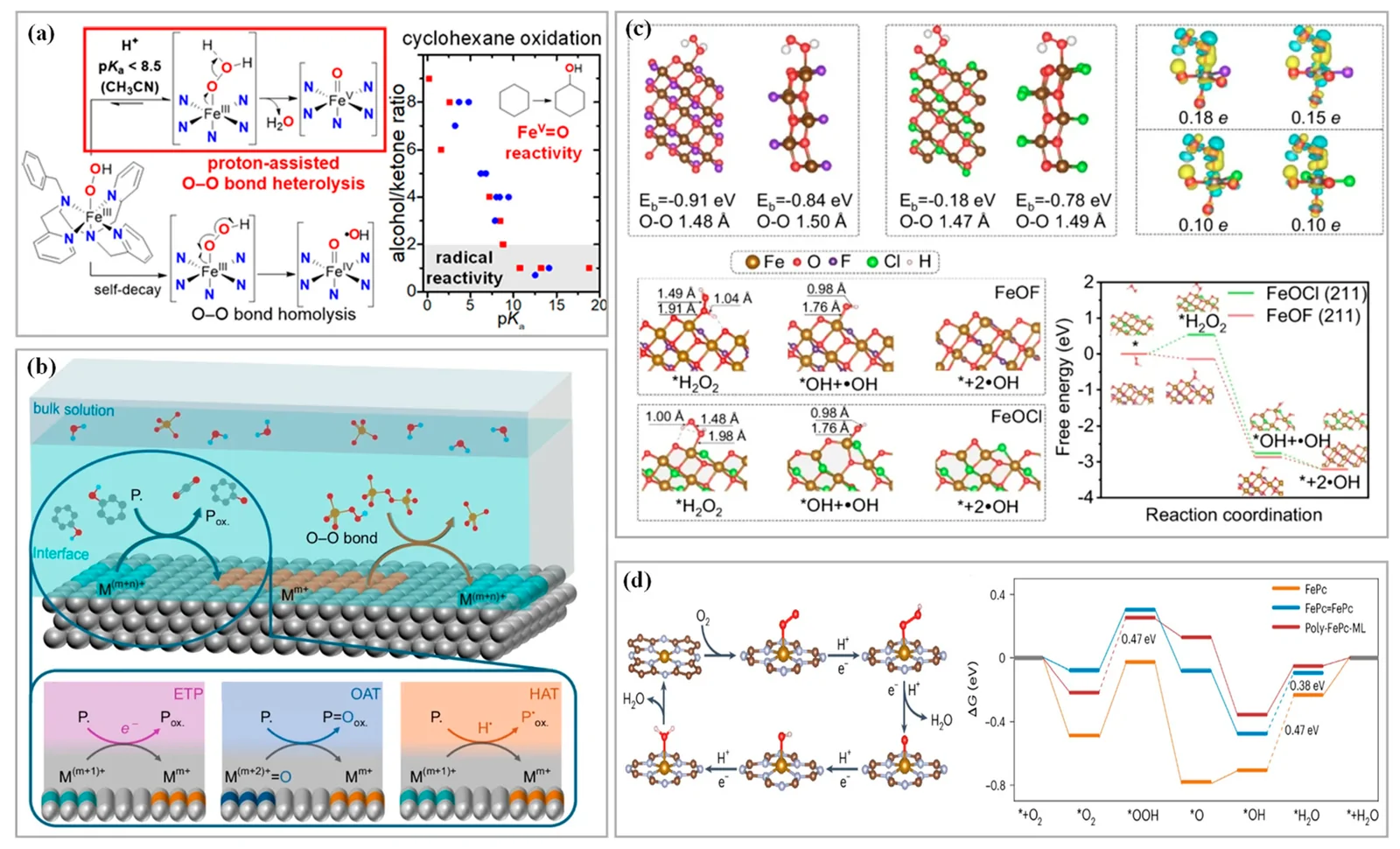
H_2_O_2_-Bound Fe-OOH Intermediates. (**a**) Proton activity controls the cleavage coordinate of Fe(III)-OOH. Reprinted with permission from Ref. [35]. Copyright 2019, American Chemical Society. (**b**) Peroxide activation couples electron motion to proton transfer at water–solid interfaces. Reprinted with permission from Ref. [49]. Copyright 2025, Springer Nature. (**c**) Axial F coordination changes H_2_O_2_ adsorption and O-O activation. Reprinted with permission from Ref. [50]. Copyright 2025, Springer Nature. (**d**) Axial OH can define a catalytically relevant Fe^3+^N_4_ state. Reprinted with permission from Ref. [51]. Copyright 2025, Springer Nature. The asterisk (*) denotes a surface site; species preceded by * are surface-bound intermediates rather than free radicals in solution.

Inner-sphere H_2_O_2_ activation begins when peroxide competes with weakly bound solvent molecules for coordination space at Fe centers. Fe-O bonding polarizes the peroxide, while electron redistribution between Fe and the O-O antibonding manifold weakens the peroxide bond. Proton transfer through water, neighboring N/O groups, or Fe-bound hydroxide can further reorganize the adduct into an Fe-OOH-type state. For this reason, the first mechanistic consequence of H_2_O_2_ adsorption is not O-O cleavage itself, but formation of a dynamically solvated Fe-peroxide coordination ensemble in which axial occupancy, hydrogen bonding, and Fe electronic structure are already reorganized (Figure 4a,b) [35,49].

Fe-OOH should be used here as an ensemble descriptor rather than a unique structural formula. This consideration is particularly important for heterogeneous Fe-N-C catalysts, where local heterogeneity prevents direct transfer of molecular Fe-OOH assignments. Its protonation state, Fe oxidation state, hydrogen-bond environment, and coordination geometry may vary with pH and support chemistry. Direct operando identification of a discrete Fe-OOH geometry on heterogeneous Fe single-atom catalysts remains substantially less developed than evidence for the products formed after peroxide cleavage. Thus, schematic representations of Fe-OOH in heterogeneous SACs should be interpreted as families of chemically related peroxide-bound states rather than spectroscopically established molecular structures.

The mechanistic significance of Fe-OOH is that it defines a branch point between one- and two-electron O-O cleavage. Molecular iron chemistry shows that proton activity and ligand field can redirect Fe(III)-OOH evolution toward homolytic or heterolytic cleavage [35]. These molecular results should not be transferred literally to heterogeneous Fe-N-C structures, but they establish a general coordination principle. O-O cleavage cannot be interpreted independently of proton transfer and ligand-field reorganization. Fe-OOH should therefore be positioned upstream of both radical formation and high-valent Fe-oxo generation rather than assigned a priori to either pathway (Figure 4c) [50]. In heterogeneous Fe-Nx systems, peroxide binding is most defensibly described as generating a family of Fe-OOH-like precursors whose fate depends on Fe valence, spin, axial accessibility, proton relays, and support-mediated electron transfer. The relevant descriptor is the population and interconversion kinetics of these states, not the stability of a single optimized adsorption geometry (Figure 4d) [51].

#### 2.2.2. Oxidant Geometry-Dependent Fe-Peroxysulfate Working States

**Figure 5:**
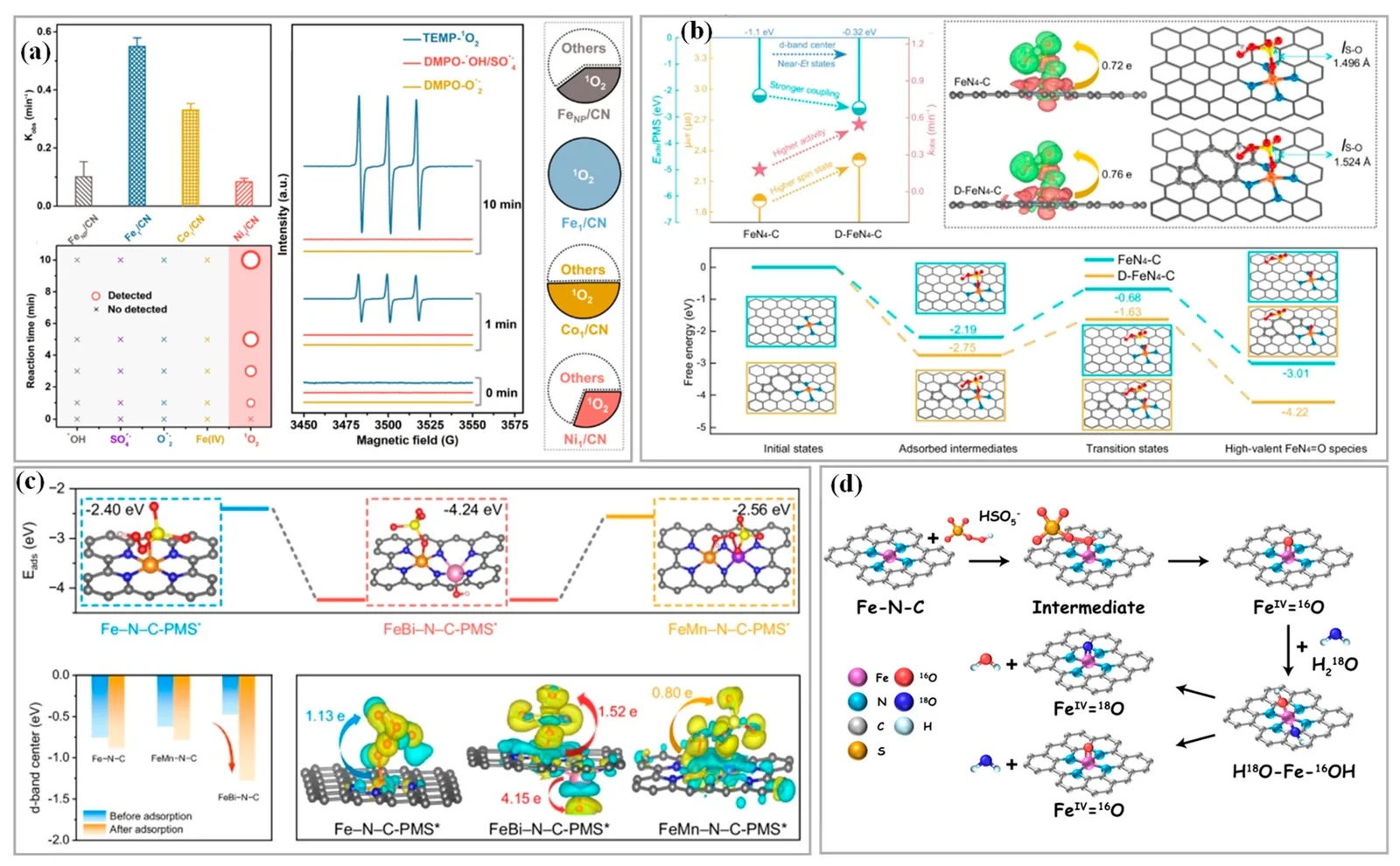
(**a**) Oxidant Geometry-Dependent Fe-Peroxysulfate Working States. Reprinted with permission from Ref. [23]. Copyright 2021, Wiley. (**b**) Long-range lattice defects tune neighboring Fe-N_4_/PMS states. Reprinted with permission from Ref. [30]. Copyright 2024, Springer Nature. (**c**) A p-block neighbor and Fe jointly polarize different PMS oxygen atoms. The asterisk (*) denotes adsorbed PMS. Reprinted with permission from Ref. [34]. Copyright 2025, Springer Nature. (**d**) Operando IR distinguishes PMS-induced Fe(IV)=O formation from alternative pathways. Reprinted with permission from Ref. [15]. Copyright 2025, Springer Nature.

PMS and PDS introduce a more complex coordination problem because the peroxide unit is coupled to sulfate. Their distinct molecular symmetry and protonation states create different coordination trajectories before electron transfer or bond cleavage occurs. PMS is chemically asymmetric and proton-bearing, whereas PDS is symmetric and dianionic. These differences affect adsorption orientation, proton transfer, O-O polarization, and sulfate-fragment release. They also alter the number and spatial arrangement of oxygen atoms capable of interacting with Fe, neighboring atoms, hydrogen-bond donors, and interfacial water, thereby creating a broader family of accessible oxidant-bound states than is typically represented by a single adsorption model (Figure 5a) [23].

Current evidence does not justify assigning a single universal molecular geometry to the surface-bound state. “Fe-peroxysulfate intermediate” is therefore preferable to an overly specific Fe-O-O-SO3 formula. The term encompasses Fe-bound PMS/PDS configurations, hydrogen-bond-stabilized structures, and cooperative adsorbate states involving neighboring Fe or other atomic centers. End-on, alternative-O-bound, hydrogen-bond-assisted, and multisite configurations should therefore be considered competing members of a peroxysulfate working-state ensemble rather than mutually exclusive structural assignments (Figure 5b) [30].

Several reaction motifs illustrate how oxidant-binding geometry governs divergence among radical, high-valent oxo, and electron-transfer pathways. Fe-N_4_/PMS systems can support terminal-O interactions associated with nonradical chemistry [23], whereas neighboring Fe sites can cooperate with an Fe-PMS* complex to increase charge transfer and promote Fe(IV)=O formation [32]. Adjacent p-block atoms provide another cooperative geometry by interacting with a second oxygen of PMS while reorganizing the electronic structure of Fe [34]. The atom that binds one part of the oxidant therefore need not perform the subsequent redox step. Coordination geometry controls not only adsorption strength but also the distribution of charge transfer, proton motion, bond polarization, and leaving-group stabilization across the catalyst–oxidant ensemble (Figure 5c,d) [15,34].

The clearest evidence that oxidant identity changes the working-state distribution comes from time-resolved measurements on the same Fe-N_4_ catalyst. Under the investigated conditions, PMS generated a detectable surface Fe(IV)=O state, whereas PDS activation exhibited predominantly radical characteristics without an observable Fe(IV)=O signature [15]. This result does not establish a universal PMS-nonradical or PDS-radical rule. It demonstrates that oxidant geometry and proton inventory reshape accessible coordination trajectories even when the resting Fe site is nominally unchanged. PMS and PDS should therefore be considered distinct coordination reagents as well as distinct oxidants because each reorganizes the working-state population before the dominant oxidation pathway emerges.

#### 2.2.3. Fe-Hydroxo/Fe-oxo Interconversion and High-Valent Working States

**Figure 6:**
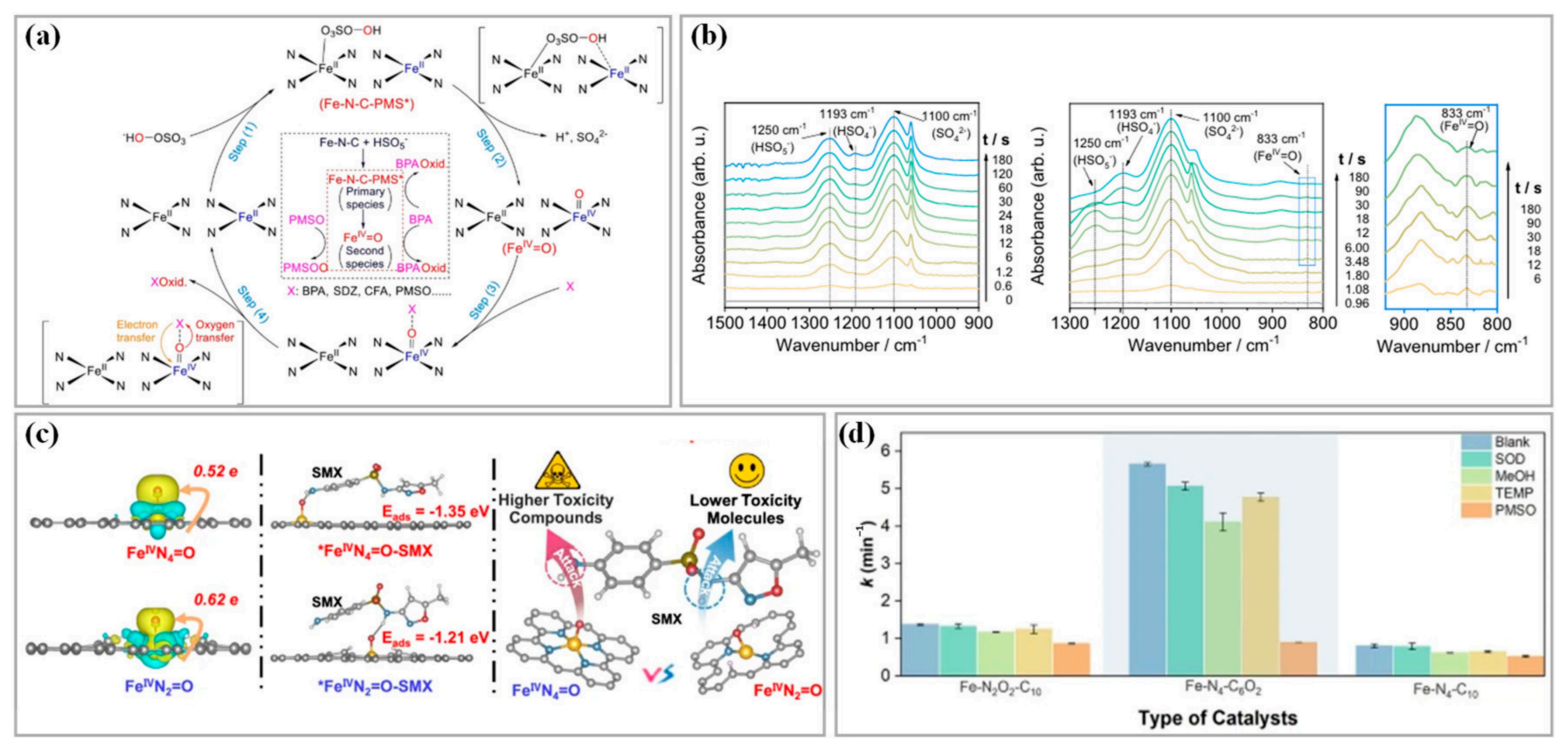
Fe-Hydroxo/Fe-oxo Interconversion and High-Valent Working States. (**a**) PMS oxygen transfer generates a kinetically competent high-valent Fe state. Reprinted with permission from Ref. [32]. Copyright 2023, Wiley. (**b**) Stopped-flow IR follows Fe(IV)=O growth, decay, and isotope response. Reprinted with permission from Ref. [15]. Copyright 2025, Springer Nature. (**c**) First-shell coordination controls how much Fe(IV)=O becomes accessible. Reprinted with permission from Ref. [25]. Copyright 2024, Springer Nature. (**d**) Remote O increases Fe=O electrophilic reactivity without weakening Fe-N_4_. Reprinted with permission from Ref. [16]. Copyright 2025, Springer Nature. The asterisk (*) denotes adsorbed PMS.

Fe-OH occupies a central position between oxidant cleavage and site regeneration. It may arise from peroxide homolysis, protonation of Fe=O, hydrolysis of an oxidized Fe center, or ligand exchange with water. Depending on proton activity and Fe electronic structure, Fe-OH can participate in proton-coupled electron transfer, regenerate a reduced site, or become a kinetically persistent species that blocks subsequent oxidant coordination. Fe-OH should therefore be viewed as a mechanistic hub connecting oxidant cleavage, high-valent-state formation, proton transfer, and site recovery rather than merely as a terminal product of peroxide decomposition (Figure 6a) [32]. High-valent Fe-oxo represents a family of oxidized working-state configurations rather than a single universally defined species. For heterogeneous Fe single-atom Fenton-like systems, Fe(IV)=O currently has the strongest direct experimental support among high-valent Fe-oxo assignments [35]. Operando stopped-flow ATR-IR resolved an Fe=O vibrational feature during PMS activation with millisecond temporal resolution and revealed a transient Fe=O feature with a measurable catalytic lifetime. Isotope experiments further demonstrated oxygen exchange between the Fe-oxo state and water, showing that solvent can enter the coordination chemistry of the working state rather than functioning only as a dielectric medium. These observations are particularly important because they establish Fe-oxo as a temporally resolved catalytic state and directly connect its oxygen coordination sphere to the surrounding aqueous environment (Figure 6b) [15].

Coordination regulates both the probability of forming Fe(IV)=O and the reactivity of the resulting state. Lower Fe-N coordination can increase Fe-oxo formation by altering electron supply and coproduct desorption [25], whereas second-shell oxygen can preserve Fe-N_4_ anchoring while tuning Fe=O frontier orbitals [16]. Adjacent p-block sites provide an additional route through cooperative electronic modulation [34]. Fe-oxo formation and Fe-oxo utilization are therefore distinct coordination problems. The former concerns access to the oxidized state, whereas the latter depends on substrate attack and intermediate turnover. A modification that increases the Fe(IV)=O population does not necessarily increase the intrinsic reactivity or productive utilization of each Fe(IV)=O state (Figure 6c,d) [16,25]. In heterogeneous Fe single-atom Fenton systems, Fe(IV)=O is currently the most strongly supported high-valent working state. Higher-valent assignments remain less firmly established and should be treated as system-specific hypotheses unless supported by direct spectroscopy, isotope response, kinetics, and theory [15]. Detecting a transient state, demonstrating its kinetic competence, and engineering the flux that it carries are separate mechanistic tasks.

Oxidant activation should therefore be viewed as a coordination-driven state transition rather than a simple electron-transfer event. H_2_O_2_, PMS, and PDS do more than supply oxidizing equivalents. They reorganize Fe coordination, redistribute electronic states, and determine which reactive intermediates become accessible.

### 2.3. Reversible Coordination Dynamics During Turnover and Irreversible Structural Transformation

Structural mobility is intrinsic to catalytic turnover, but structural change is not synonymous with dynamic catalysis. Productive dynamics comprise reversible changes in valence, spin, axial ligation, Fe position, and ligand distance that occur within a catalytically connected coordination network and contribute to oxidant activation, substrate conversion, or site regeneration. Demetallation, migration, aggregation, and irreversible support degradation instead represent escape from that network. Reversibility is therefore necessary but not sufficient. The structural change must also correlate with turnover and preserve active-site identity. A working-state population should respond to the reaction driving force and return toward its resting distribution when oxidant or potential is removed. Irreversible transformation produces cumulative loss or replacement of the original Fe site even when transient catalytic activity persists.

#### 2.3.1. Valence, Spin, and Geometric Adaptation

**Figure 7:**
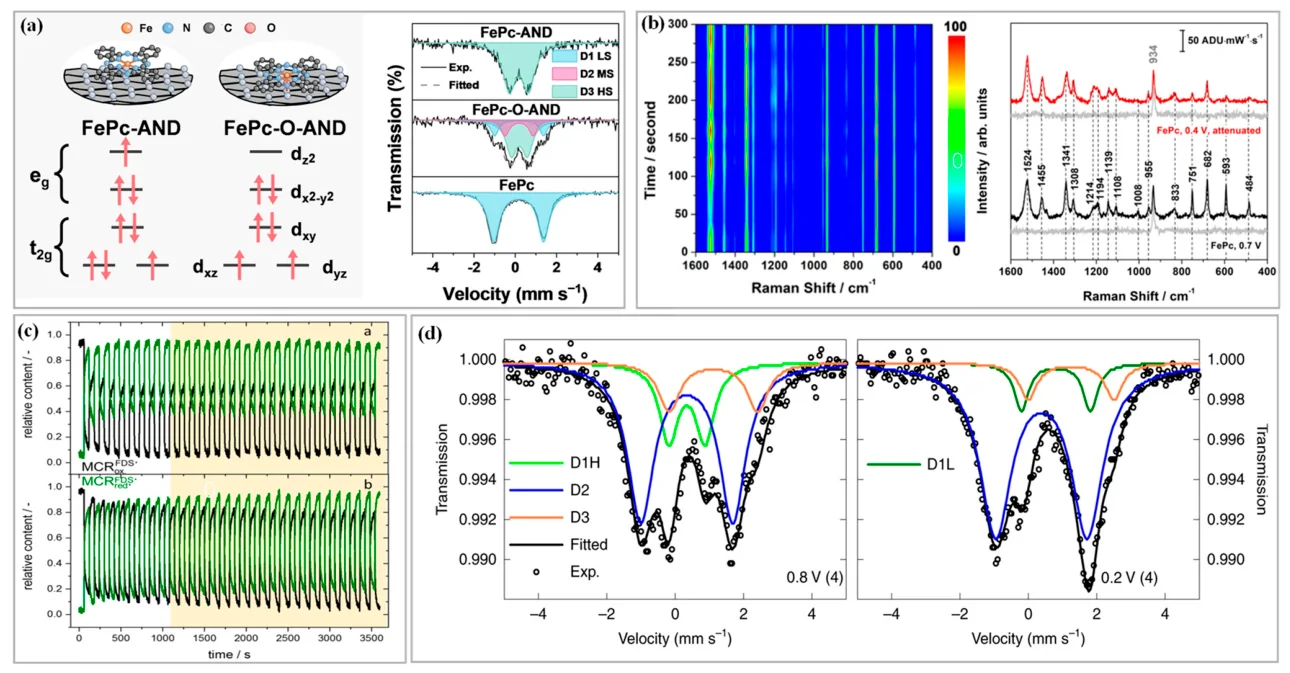
Reversible adaptation and deactivation of Fe single-atom sites. (**a**) Axial-O-dependent electronic and spin-state changes; reproduced from Ref. [48]. (**b**) Operando Raman observation of FeN_4_ distortion and subsequent demetallation; reproduced from Ref. [29]. (**c**) Multivariate-curve-resolution concentration profiles of Fe-N-C components under potential modulation during oxygen reduction, included as a methodological analogy rather than direct AOP evidence; reproduced from Ref. [53]. (**d**) Mössbauer-resolved evolution of FeN_4_ populations; reproduced from Ref. [28]. Reprinted with permission from Ref. [48] (Copyright 2026, Springer Nature), [29] (Copyright 2019, American Chemical Society), [53] (Copyright 2022, Wiley), and [28] (Copyright 2021, Springer Nature), respectively.

Fe valence changes during oxidant activation are coupled to ligand binding and coordination rearrangement. Fe(II)/Fe(III) interconversion commonly accompanies one-electron peroxide chemistry, whereas Fe(IV)=O can arise after oxygen-atom transfer or heterolytic cleavage. The catalytic relevance of these changes lies not in formal oxidation state alone but in whether the redox transition belongs to a closed catalytic cycle. Spin provides an independent degree of freedom because identical coordination formulas can represent electronically distinct states. Mössbauer studies show that FeN_4_ populations with similar first-shell compositions can exhibit different spin states and markedly different durability under operation [27,28,52]. Axial oxygen coordination can further induce or stabilize a spin transition by reorganizing the 3dz^2^ orbital and the electronic accessibility of the axial reaction coordinate (Figure 7a) [48].

Electronic adaptation is accompanied by geometric motion. Axial binding can displace Fe from the N_4_ plane and alter equatorial bond lengths. Operando tip-enhanced Raman spectroscopy on Fe phthalocyanine directly distinguished reversible nonplanar deformation from subsequent irreversible demetallation (Figure 7b) [29]. Out-of-plane displacement is therefore not itself a degradation marker. Instead, it represents an adaptive geometric response whose catalytic significance depends on reversibility and electronic consequence. Claims of local support bending or large Fe-N excursions require additional caution. Calculated structures can establish mechanical plausibility, but dynamic geometric distortions should not be presented as directly observed unless supported by appropriate operando structural or vibrational data. This distinction is essential because theoretical predictions often reveal possible distortions, whereas experimental validation requires evidence that such distortions occur under catalytic conditions.

#### 2.3.2. Ligand Exchange and Recovery of the Resting State

Aqueous Fe single-atom sites are rarely coordinatively bare. The solvated resting state should therefore be considered a hydrated coordination ensemble rather than the dehydrated structure obtained after synthesis or vacuum characterization. Water and OH^−^ can occupy axial positions even before oxidant exposure, meaning that the true solution-phase resting state may differ from the dry structure obtained by ex situ spectroscopy. Oxidant activation then involves ligand exchange within this pre-existing solvent shell. H_2_O_2_, PMS, or PDS may displace water, bind to an Fe-OH species, or occupy a transiently available coordination position. After O-O activation, the oxidant ligand is replaced by hydroxo, oxo, sulfate-derived, or product-bound states [28]. The lifetime of these substituted states determines whether coordination exchange supports turnover or induces site blockage. Regeneration requires these ligands to be removed through proton transfer, electron transfer, hydrolysis, or displacement by water. The essential kinetic requirement is not weak adsorption, but reversible occupancy with balanced binding strength that enables activation without preventing product release. An oxidant must remain bound long enough to activate, while products and coproducts must depart fast enough to reopen the site. Excessively strong sulfate or hydroxide binding can therefore suppress turnover even when the initial peroxide activation step is favorable.

Temporary weakening of an Fe-N bond should not be equated with permanent conversion of Fe-N_4_ to Fe-N_3_. In operando XAS, an apparent coordination change can also reflect bond-length disorder rather than ligand loss. Recovery of the initial signature and preservation of the active-site population across repeated cycles are therefore required to support reversible reconstruction. The concentration profiles in Figure 7c originate from multivariate curve resolution of time-resolved modulation–excitation XAS during Fe-N-C oxygen reduction [53]. They trace the relative amounts of fitted spectral components under potential modulation; they are not XAS spectra and do not establish an analogous Fe-peroxide intermediate in AOPs. The transferable methodological lesson is to test whether a state-dependent signal follows a controlled perturbation, returns when the perturbation is reversed, and remains reproducible without progressive site loss. In peroxide oxidation, those criteria require Fe-sensitive measurements under the relevant oxidant, substrate, and aqueous conditions.

#### 2.3.3. Deactivation Pathways, Demetallation, Migration, and Aggregation

Irreversible Fe-ligand cleavage marks the principal boundary between adaptive coordination dynamics and catalyst failure. Once Fe no longer returns to its original coordination cavity, the site has left the productive cycle. Protonation of coordinating N, oxidation of adjacent carbon, repeated high-valent cycling, or excessive axial pulling can all promote this escape. Demetallation and leaching should be distinguished. Demetallation is loss of Fe from its original ligand environment; leaching is transfer of that Fe into solution. An Fe atom can demetallate and remain trapped as an oxide or cluster, while dissolved Fe may originate from pre-existing particles rather than an active FeN_x_ population. Consequently, activity loss or bulk Fe analysis alone cannot identify the degradation pathway.

Operando Mössbauer measurements demonstrate that distinct FeN_4_ populations can exhibit markedly different durability, with unstable sites converting into iron-oxide species while other FeN_4_ environments remain structurally conserved (Figure 7d) [28]. Molecular-scale Raman measurements likewise resolve direct demetallation of FePc after an earlier reversible geometric response [29]. These studies establish that reversible distortion and irreversible metal loss can occur sequentially and must be experimentally separated. Migration and aggregation require evidence beyond loss of the original Fe-N signature. New Fe-Fe scattering, altered Mössbauer populations, spatially resolved microscopy, or equivalent structural evidence is required to establish atom pairing or cluster formation. Similarly, retention of total Fe does not demonstrate retention of single-atom sites. The disappearance of a single-atom spectroscopic signature should therefore not automatically be interpreted as aggregation without direct evidence of Fe-Fe interaction or nanoscale redistribution. Support degradation represents a coupled failure mode. Oxidation of the carbon or molecular framework can remove coordinating atoms, enlarge defects, and increase Fe mobility. Once this process changes the local electronic and transport environment irreversibly, preservation of some Fe atoms is insufficient to claim preservation of the original catalyst. Only reversible changes that participate in catalytic turnover should therefore be defined as productive dynamic coordination. Demetallation, migration, aggregation, and support collapse should be classified as deactivation trajectories, even when newly generated species retain temporary catalytic activity.

Collectively, dynamic coordination should not be evaluated by structural change alone but by the coupling between structural reversibility, catalytic function, and active-site preservation. During turnover, Fe sites continuously adapt their valence, geometry, and ligand environment to accommodate oxidant activation and substrate transformation. The same flexibility, however, becomes detrimental when coordination recovery fails and irreversible pathways dominate. Distinguishing productive adaptation from structural degradation is therefore essential for understanding catalyst lifetime and designing durable Fe single-atom catalysts.

## 3. Coordination-Governed Catalytic Pathway Evolution

### 3.1. Coordination-Controlled Divergence of Radical and Fe-oxo Pathways in H_2_O_2_ Activation

H_2_O_2_ provides a fundamental peroxide platform for understanding how Fe coordination controls O-O activation and subsequent pathway divergence. After inner-sphere binding, the Fe-peroxide working state can evolve through one-electron cleavage toward radical chemistry or through more strongly polarized, proton-coupled cleavage toward high-valent Fe-oxo states. These pathways are connected by the same redox inventory and compete for the same Fe coordination space.

#### 3.1.1. Homolytic O-O Cleavage and Radical Formation

Classical Fenton chemistry couples H_2_O_2_ reduction to oxidation of a reduced Fe center. In heterogeneous Fe single-atom catalysts, however, this elementary step occurs within a constrained coordination environment where ligand exchange and electron redistribution jointly determine the cleavage mode. In an Fe-bound peroxide ensemble, one-electron transfer into the O-O antibonding manifold promotes cleavage and generates Fe-OH together with a hydroxyl-radical equivalent. The probability of this route depends on electron availability at Fe, axial accessibility, proton organization, and the ease with which the resulting Fe state is regenerated.

**Figure 8:**
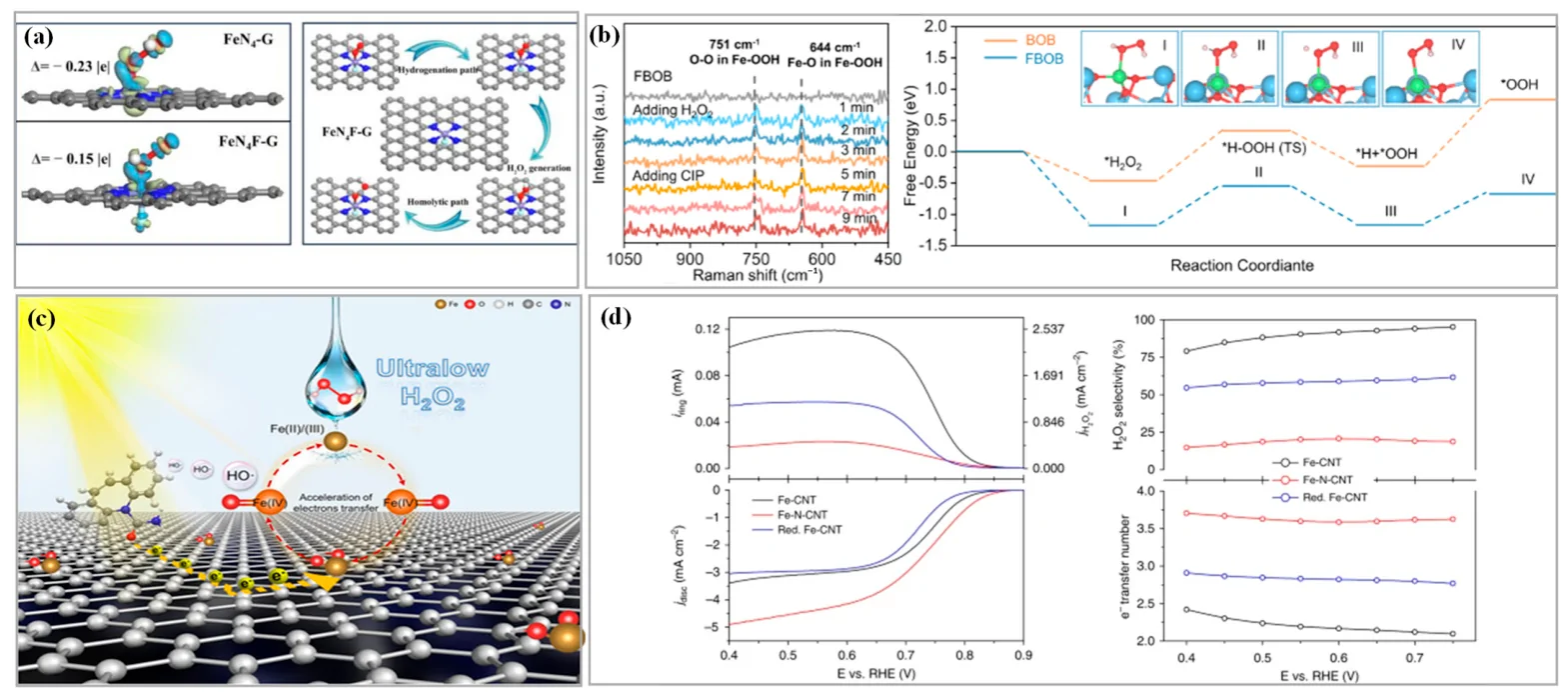
Coordination-controlled pathway divergence and Fe-site regeneration during H_2_O_2_ activation. (**a**) Axial F coordination regulates H_2_O_2_ adsorption and ^•^OH generation at FeN_4_F single-atom sites. Reprinted with permission from Ref. [54]. Copyright 2026, Wiley. (**b**) In situ Raman identification of Fe-*OOH and the corresponding H_2_O_2_ activation pathway toward O_2_•− over high-spin Fe sites. The asterisk (*) denotes a surface site. Reprinted with permission from Ref. [52]. Copyright 2024, National Academy of Sciences. (**c**) H_2_O_2_-triggered Fe(IV)=O formation, pollutant-to-Fe(IV)=O electron transfer, and light-assisted H_2_O_2_ regeneration over Fe1-GO. Reprinted with permission from Ref. [55]. Copyright 2026, Wiley. (**d**) Coordination-dependent oxygen-reduction selectivity of Fe-C-O and Fe-C-N motifs toward H_2_O_2_ and H_2_O, respectively. Reprinted with permission from Ref. [56]. Copyright 2019, Springer Nature.

Coordination can redirect both the identity and flux of radical species generated during peroxide activation. Electron withdrawal by fluorine coordination modifies H_2_O_2_ adsorption and O-O activation at Fe centers (Figure 8a) [54], whereas high-spin Fe environments have been shown to favor H_2_O_2_-derived O_2_^•−^ under neutral and alkaline conditions (Figure 8b) [52]. The latter result is mechanistically important because it demonstrates that “radical Fenton chemistry” is not synonymous with free ^•^OH production. Spin-dependent electron transfer can shift the radical manifold while the oxidant remains H_2_O_2_. The commonly used terminology therefore requires caution when comparing heterogeneous systems with classical homogeneous Fenton chemistry.

Spatial location further determines the effective chemistry of short-lived radicals. Species escaping into bulk water undergo nearly diffusion-controlled competition with carbonate, natural organic matter, excess peroxide, and other solutes [22]. Radical equivalents generated close to an adsorbed pollutant or within a confined hydration layer can react before diffusional escape. Such behavior should be described cautiously; “surface-confined ^•^OH” may represent an adsorbed radical, an Fe-OH state with hydroxyl-like reactivity, or simply ^•^OH generated within a diffusion distance shorter than the characteristic escape length. Such distinctions are essential because apparent radical pathways may originate from interfacial Fe-OH chemistry rather than freely diffusing radicals.

The radical branch is sustained only if the oxidized Fe state is returned to an H_2_O_2_-accessible configuration. Accumulation of Fe(III)-OH, strongly bound products, or oxidized support domains can reduce radical flux even while H_2_O_2_ remains available. The rate of radical production is therefore controlled by the complete Fe redox-coordination loop, not by the O-O-cleavage step alone.

#### 3.1.2. Heterolytic Cleavage and Fe-oxo Formation

An alternative reaction coordinate retains the oxidizing equivalent at Fe. This pathway converts oxidant activation from a diffusional radical process into a metal-centered oxygen-transfer cycle. Strong polarization of an Fe-OOH-type state, coupled to proton transfer, can favor heterolytic O-O cleavage and formation of high-valent Fe-oxo. Unlike free radicals, Fe-oxo remains electronically and spatially coupled to the coordination environment, allowing ligand field, spin, substrate orientation, and local proton activity to determine its reactivity. H_2_O_2_-driven Fe-oxo chemistry is not restricted to coordinatively unsaturated Fe. In molecularly defined or heterogeneous oxidation models, Fe-N_4_ configurations supported selective benzene hydroxylation through Fe=O/O=Fe=O-type intermediates, whereas progressive replacement of N ligands lowered activity [22]. Likewise, different N environments around Fe altered Fe-oxo accessibility despite apparently favorable Fe(II) populations in the less active structures [24]. These observations demonstrate that Fe valence alone is an insufficient descriptor.

Proton transfer provides an additional control coordinate. Recent heterogeneous peroxide studies show that electron transfer at a solid–water interface can be strongly coupled to proton motion rather than proceeding as an isolated electronic event [49,57]. Although these studies extend beyond Fe single-atom H_2_O_2_ systems, they establish a general constraint relevant to Fe-OOH cleavage. The energetic cost of proton transfer can be comparable to that of electron transfer. Local proton relays and surface O-H bond energetics should therefore be included explicitly in the reaction coordinate.

Once formed, Fe-oxo can react through oxygen-atom transfer, hydrogen-atom abstraction, electrophilic addition, or electron transfer. The preferred elementary step depends on oxo electrophilicity, spin state, substrate bond strength, oxidation potential, and adsorption geometry. An Fe1/graphene-oxide system illustrates this distinction. H_2_O_2_-induced Fe(IV)=O promoted electron transfer from carbamazepine rather than behaving as a freely diffusing oxidant (Figure 8c) [55]. An Fe-oxo pathway thus identifies a working-state manifold rather than a single substrate-oxidation mechanism. Radical and Fe-oxo branches should be considered competing exits from an Fe-peroxide precursor. Greater one-electron-transfer propensity favors radical cleavage, whereas organized proton transfer and stronger peroxide polarization can facilitate high-valent Fe formation. Their relative contributions can evolve during turnover as Fe valence, surface hydroxylation, substrate coverage, and local proton activity change.

#### 3.1.3. Fe-Site Regeneration and Redox Supply Under Thermal, Photochemical, and Electrochemical Conditions

Working-state formation generates activity, whereas regeneration determines whether that activity persists. After radical formation, oxidized Fe must regain electron density. After Fe-oxo-mediated oxidation, Fe-OH or another product-bound state must undergo reduction, proton transfer, ligand release, and water recoordination before H_2_O_2_ can re-enter the cycle. Pathway efficiency is therefore determined by closure of the entire Fe coordination cycle rather than by intermediate formation alone. Under dark thermal conditions, heat accelerates charge transfer, ligand exchange, and desorption but does not supply reducing equivalents. Regeneration must instead be coupled to H_2_O_2_ chemistry, substrate-derived reductants, support redox states, or neighboring electron reservoirs. Rapid initial H_2_O_2_ activation can therefore coexist with poor sustained turnover when the reduced Fe population is regenerated slowly.

Photochemical operation supplies a distinct electron reservoir. In Fe1-graphene oxide, visible light couples substrate-to-Fe(IV)=O electron transfer with O_2_ reduction and H_2_O_2_ replenishment, generating a feedback loop between oxidant consumption and production in that specific architecture [55]. Light therefore alters both the Fe redox cycle and the oxidant inventory. Electrochemical systems decouple electron supply more explicitly. Fe coordination can favor two-electron oxygen reduction to H_2_O_2_ over four-electron reduction to water. Fe-C-O motifs, for example, exhibit high H_2_O_2_ selectivity under oxygen-reduction conditions (Figure 8d) [56]. Coupled electro-Fenton architectures can consequently integrate H_2_O_2_ generation and Fe-mediated activation in space and time. Thermal, photochemical, and electrochemical Fenton systems differ primarily in the source of reducing equivalents and the route by which the Fe coordination environment is restored. All require regeneration of an Fe state that can rebind H_2_O_2_ without accumulating hydroxo ligands, products, or irreversibly oxidized structures.

Collectively, H_2_O_2_ activation illustrates that radical and Fe-oxo pathways are not independent reaction classes but competing trajectories originating from Fe-peroxide working states. Coordination environment determines oxidant binding geometry, electron-transfer probability, proton-coupled rearrangement, and regeneration kinetics, thereby controlling which oxidation pathway dominates under specific conditions. This coordination-governed pathway divergence provides a mechanistic basis for understanding more complex PMS/PDS activation systems discussed below.

### 3.2. Coordination-Controlled Divergence of PMS/PDS Activation Pathways

PMS and PDS expand the reaction network because peroxide cleavage is coupled to sulfate chemistry, introducing additional coordination configurations, proton-transfer steps, and competing electron-transfer channels. PMS is asymmetric and proton-bearing; PDS is symmetric, more extended, and dianionic. Their distinct geometries and proton inventories change the energetic relationship among electron transfer, proton transfer, O-O cleavage, and sulfate-fragment release.

#### 3.2.1. Radical Pathways Enabled by Fe-Peroxysulfate Electron Transfer

**Figure 9:**
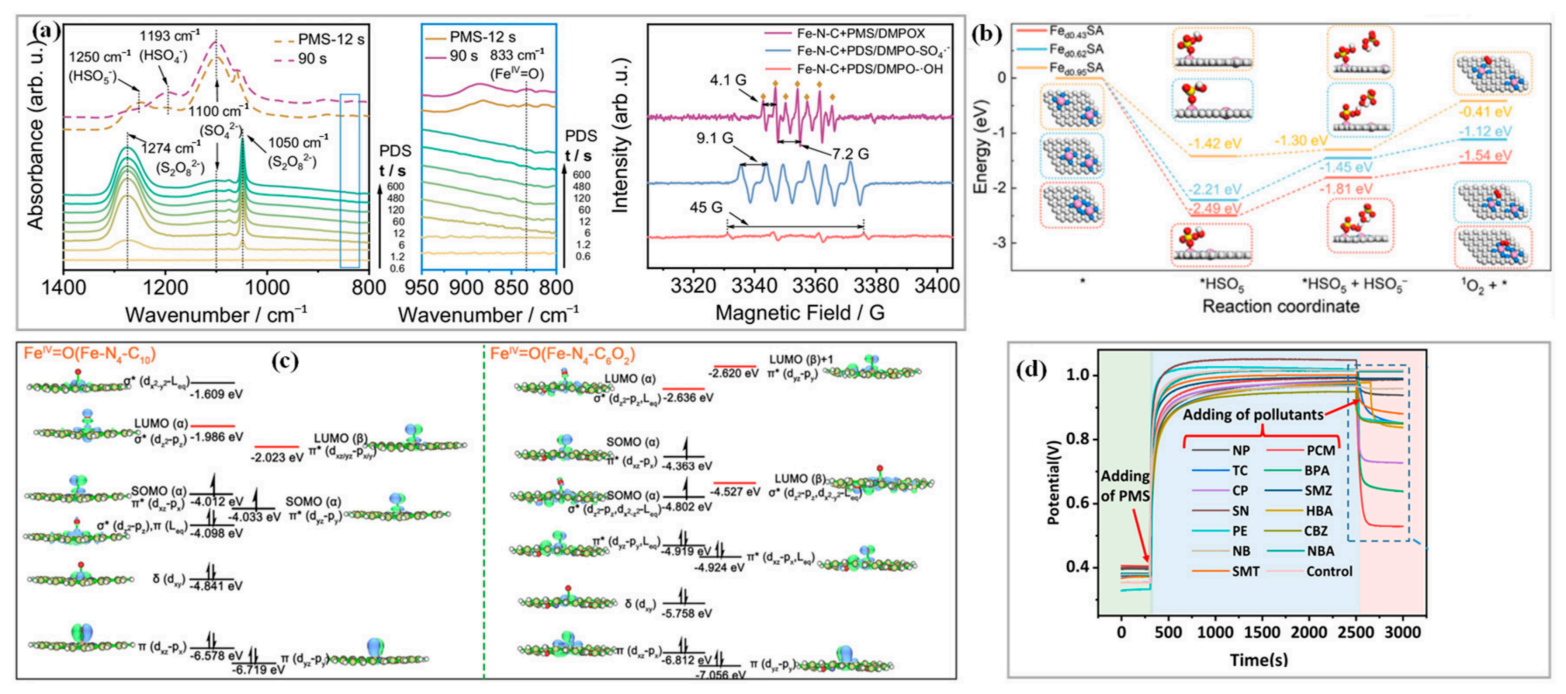
Coordination-controlled divergence of PMS/PDS activation pathways at Fe single-atom sites. (**a**) Oxidant-dependent pathway divergence on the same Fe-N_4_ catalyst. PDS predominantly generates SO_4_^•−^/^•^OH, whereas PMS produces a spectroscopically detectable Fe(IV)=O working state. Reprinted with permission from Ref. [15]. Copyright 2025, Springer Nature. (**b**) Second-shell O modulation in Fe-N_4_-C_6_O_2_ lowers the Fe=O acceptor-orbital energy and enhances the intrinsic electrophilic reactivity of Fe(IV)=O. The asterisk (*) denotes a surface site. Reprinted with permission from Ref. [16]. Copyright 2025, Springer Nature. (**c**) Proximity-regulated Fe-N_4_ twin sites in which an intermediate-spin configuration at an Fe-Fe distance of 0.43 nm favors PMS adsorption and coupling toward selective ^1^O_2_ generation. Reprinted with permission from Ref. [58]. Copyright 2026, Wiley. (**d**) Pollutant-dependent direct electron transfer through Fe-SAC/PMS surface complexes, governed by pollutant electron-donating ability and the interfacial energy-level gap. Reprinted with permission from Ref. [12]. Copyright 2024, National Academy of Sciences.

Radical activation begins when one-electron transfer into the peroxysulfate O-O antibonding manifold generates SO_4_^•−^. Subsequent aqueous reactions can convert SO_4_^•−^ into ^•^OH or other radicals depending on pH and solution composition. The apparent radical inventory therefore reflects both primary Fe-mediated cleavage and secondary solution chemistry. Operando measurements on the same Fe-N_4_ catalyst provide a particularly instructive comparison. PDS produced predominantly SO_4_^•−^/^•^OH chemistry, whereas PMS generated a detectable Fe(IV)=O working state under the investigated conditions (Figure 9a) [15]. Because the resting Fe structure was unchanged, the contrast shows that oxidant geometry can redirect catalytic flux by changing the accessible working-state distribution. It should not be generalized into a universal PDS-radical or PMS-nonradical rule. Fe electronic structure, spin, proton relays, binding orientation, and the free-energy cost of sulfate release all influence the branch [46,52]. Both oxidants can therefore access overlapping mechanistic manifolds. The sustained radical flux also depends on spatial utilization and Fe-site regeneration. Rapid O-O cleavage coupled to slow Fe reduction produces only a transient radical burst rather than a closed catalytic cycle.

#### 3.2.2. High-Valent Fe-oxo Pathways Enabled by Electronic Modulation

PMS can transfer an oxygen atom to Fe without first releasing a free radical. Operando stopped-flow spectroscopy provides the strongest direct evidence for this branch by resolving a surface Fe(IV)=O vibration, its isotope response, and a lifetime of several seconds during Fe-N_4_/PMS turnover [15]. This lifetime is sufficient for Fe(IV)=O to act as a kinetically competent oxidant rather than an unresolved transition-state structure. Two quantities must nevertheless be separated. One is the accessibility of Fe-oxo formation, and the other is the intrinsic oxidation capability of the Fe-oxo state. Lower Fe-N coordination can favor formation. In matched Fe-N_2_, Fe-N_3_, and Fe-N_4_ catalysts, Fe-N_2_ produced a larger Fe(IV)=O population because its altered coordination environment facilitated PMS transformation and removal of sulfate-derived intermediates [25].

Remote coordination can instead regulate the intrinsic reactivity of Fe-oxo. Second-shell O in Fe-N_4_-C_6_O_2_ preserves the primary Fe-N_4_ structure while lowering the Fe=O acceptor-orbital energy and increasing electrophilic reactivity (Figure 9b) [16]. A catalyst can therefore improve Fe-oxo chemistry without increasing the steady-state Fe-oxo population. Cooperative sites provide additional routes to the same working state. Neighboring p-block atoms can interact with PMS while electronically modulating Fe, thereby increasing the Fe(IV)=O population and shifting an electron-transfer-dominated regime toward high-valent Fe oxidation [34]. An Fe-N-C-PMS* state can likewise receive additional charge from a nearby Fe site and evolve toward Fe(IV)=O [32]. The surrounding coordination network, rather than the first oxidant-binding atom alone, determines whether activation proceeds through radical dissociation, oxygen transfer, or interfacial electron transfer. Productive turnover then requires substrate attack, sulfate-fragment release, Fe-OH conversion, and restoration of axial accessibility. Fe-oxo formation, utilization, and regeneration should therefore be evaluated as separate kinetic processes.

#### 3.2.3. Second-Shell-Regulated Singlet Oxygen and Direct Electron-Transfer Pathways

PMS can also support nonradical pathways without a dominant Fe-oxo population. Fe-N_4_ supported on carbon nitride has been reported to direct PMS conversion toward ^1^O_2_ [23], demonstrating that the nominal Fe-N_4_ motif need not converge on either free-radical cleavage or metal-oxo formation. More recent proximity engineering provides a stronger structural link between the Fe microenvironment and ^1^O_2_ generation. Precisely controlled neighboring Fe-N_4_ sites alter ligand-field strength and spin. An intermediate-spin configuration at the optimized intersite distance favors PMS adsorption and coupling associated with selective ^1^O_2_ formation (Figure 9c) [58]. This result extends pathway regulation from a single first shell to intersite distance and spin coupling. The assignment of ^1^O_2_ nevertheless requires caution. D_2_O enhancement, TEMP-EPR signals, and furfuryl-alcohol inhibition are not independently definitive, and quantitative analyses show that ^1^O_2_ contributions in PMS systems can be overestimated [59]. A convincing assignment should demonstrate that the measured ^1^O_2_ concentration and second-order substrate reactivity can account quantitatively for the observed oxidation rate after competing Fe-oxo, radical, and electron-transfer pathways are excluded.

Surface electron transfer is another nonradical pathway that competes with metal-centered oxidation. PMS remains electronically coupled to the catalyst as an activated complex, and the pollutant donates electrons directly to the catalyst–oxidant ensemble. Across single-atom/PMS systems, pollutant reactivity correlates with electronic descriptors such as electrophilicity and the interfacial energy relationship between the pollutant and catalyst–PMS complex (Figure 9d) [12]. The substrate therefore helps determine whether the activated complex is consumed before O-O cleavage. An Fe-PMS* state may undergo direct electron transfer with the pollutant or evolve through coordination rearrangement and electron redistribution toward Fe(IV)=O. The relative rates of these exits determine the observed mechanism [32,34]. Electron-transfer and Fe-oxo pathways should therefore be treated as competing outcomes of a shared oxidant-bound ensemble rather than unrelated categories.

Collectively, PMS/PDS activation demonstrates that oxidant identity alone does not dictate the oxidation pathway. Instead, PMS/PDS molecules interact with Fe coordination environments to generate diverse oxidant-bound working states, from which radical cleavage, Fe-oxo formation, singlet oxygen generation, and direct electron-transfer pathways emerge as competing kinetic exits. First-shell coordination, second-shell electronic modulation, and neighboring-site cooperation collectively determine the population and lifetime of these states, thereby governing the dominant oxidation route under practical conditions.

### 3.3. Dynamic Pathway Selection Governed by Catalyst–Oxidant–Pollutant Coupling

The preceding cycles show that pathway selection cannot be assigned from Fe structure alone. Coordination determines which working states are accessible, and the oxidant defines the initial activation landscape. Pollutant molecules and the solvent environment then determine which accessible states are formed, consumed, and regenerated most rapidly during turnover. The operative mechanism is therefore an emergent property of the catalyst–oxidant–pollutant–water ensemble rather than an intrinsic attribute of an isolated Fe site.

#### 3.3.1. Coordination-Controlled Branching of Oxidation Pathways

**Figure 10:**
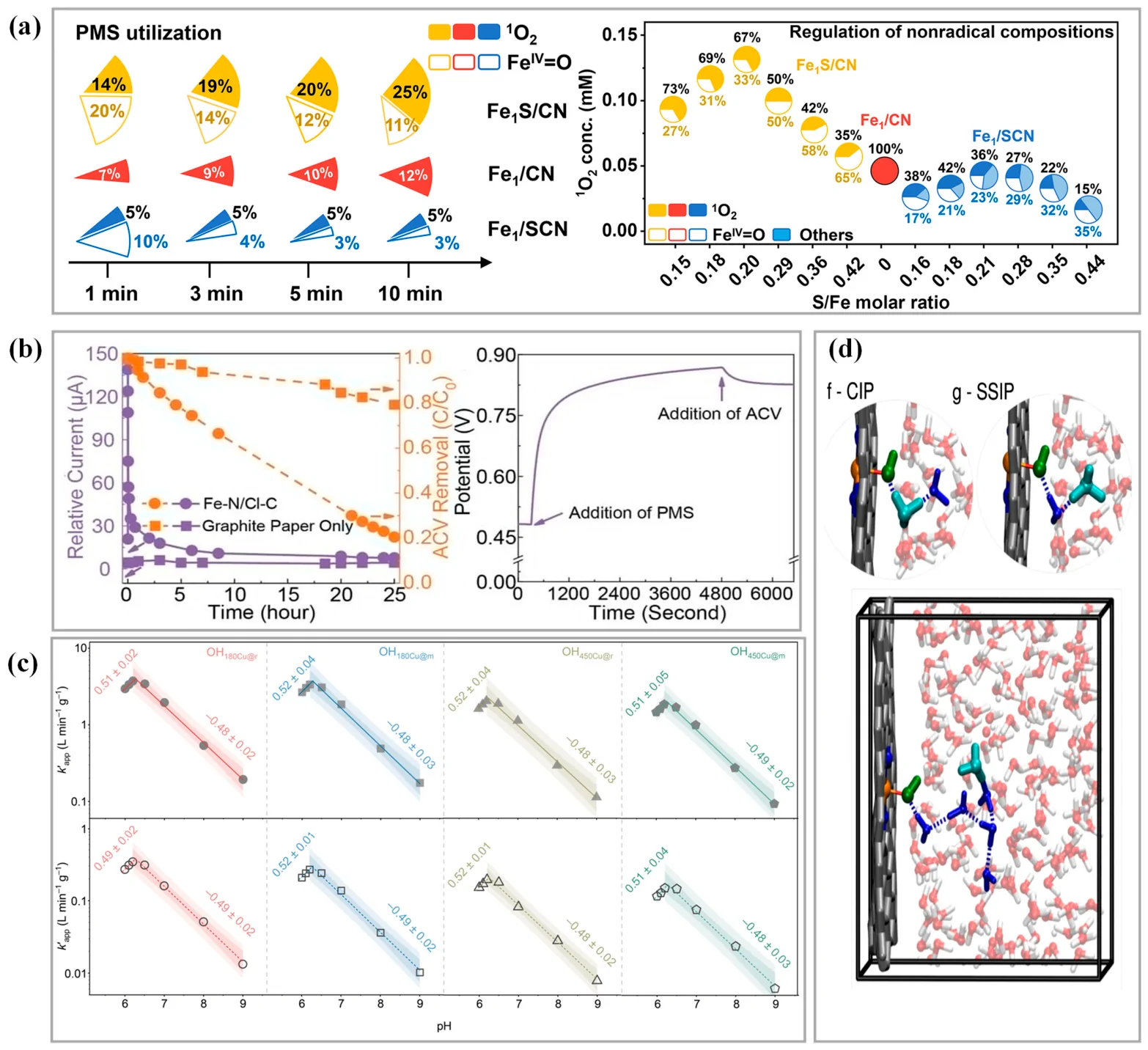
Dynamic pathway selection through catalyst–oxidant–pollutant and solvent coupling. (**a**) Spatial Fe-S coordination alters PMS binding and redistributes radical, ^1^O_2_, and Fe(IV)=O pathway flux. Reprinted with permission from Ref. [61]. Copyright 2024, National Academy of Sciences. (**b**) Pollutant adsorption and frontier-orbital energetics jointly regulate electron transfer through an Fe-N_4_-Cl/PMS* complex. Reprinted with permission from Ref. [62]. Copyright 2025, Wiley. (**c**) Surface O-H bond thermochemistry and proton activity govern proton-coupled electron-transfer kinetics. Reprinted with permission from Ref. [57]. Copyright 2026, National Academy of Sciences. (**d**) Explicit interfacial water coordination and hydrogen-bond-mediated proton transfer differentiate the hydrated working states of porphyrinic and pyridinic Fe-N_4_ sites. Reprinted with permission from Ref. [63]. Copyright 2023, Springer Nature.

Coordination perturbations redirect pathway flux when they differentially stabilize elementary steps that compete after oxidant binding. Coordination engineering should therefore be evaluated by its ability to reorder the kinetics of competing pathways rather than by activity enhancement alone [25,31,48,60]. First-shell ligation, spin state, axial accessibility, higher-shell polarization, and confinement can shift the free energies of charge transfer, O-O cleavage, substrate attack, coproduct release, and site regeneration (Figure 10a) [61]. These factors operate hierarchically. First-shell ligands establish the primary electronic structure, whereas the extended microenvironment regulates interfacial dynamics. Their mechanistic value lies in changing the kinetic ordering of exits from a shared oxidant-bound ensemble rather than creating a universally more active Fe center.

This perspective also explains why isolated adsorption energies are weak universal descriptors. Strong oxidant binding is useful only when subsequent bond cleavage, substrate reaction, sulfate/hydroxide release, and Fe regeneration remain favorable. An intermediate stabilized beyond the optimal residence time may even suppress turnover by blocking coordination space required for subsequent catalytic steps. The dominant pathway is the branch that sustains the largest catalytic flux across the complete cycle, not necessarily the branch containing the most stable intermediate or the lowest single-step barrier.

#### 3.3.2. Pollutant-Regulated Electron Transfer and Pathway Selectivity

The pollutant is an active component of pathway selection rather than merely the terminal substrate. Its electron-donating ability and adsorption geometry determine whether electron transfer can compete with oxidant decomposition or formation of another reactive intermediate. A systematic study across pollutants and single-atom/PMS catalysts showed that pathway contributions vary with pollutant electronic structure, with electron-transfer oxidation becoming more important for favorable electron donors [12]. A catalyst should therefore not be classified as radical or nonradical using a single probe compound because the dominant pathway can change with substrate identity even when catalyst and oxidant remain unchanged. Electronic descriptors also require interfacial interpretation. Gas-phase ionization energy, electrophilicity, and frontier-orbital energies may provide qualitative correlations, but the relevant quantity in water is the effective interfacial electron-transfer free energy. Protonation, solvation, adsorption strength, and orbital orientation can substantially alter the energetic relationship between a pollutant and an Fe-oxidant complex.

Functional groups also control spatial coupling. Phenolic, amino, sulfur-containing, and other electron-rich groups may donate electrons while anchoring the substrate near the active site. Conversely, a molecule with a favorable bulk oxidation potential may react slowly when its adsorption geometry prevents effective orbital overlap. Adsorption configuration and electronic coupling should therefore be evaluated together (Figure 10b) [62]. The same principle applies to Fe-oxo chemistry. A pollutant that reacts rapidly with Fe=O can kinetically drain the Fe-oxo branch, whereas a strong electron donor positioned near Fe-PMS* may intercept the precursor before Fe=O forms. Catalyst coordination determines which oxidant-bound states are accessible, and pollutant chemistry determines how rapidly and selectively each state is consumed.

#### 3.3.3. Interfacial Solvation, Proton Dynamics, and Water-Matrix Effects

Water is an integral component of the Fe coordination cycle rather than a passive solvent. It can occupy axial positions, reorganize hydrogen-bond networks, relay protons, and exchange oxygen with Fe-oxo intermediates [15]. Proton activity consequently regulates peroxide protonation, Fe-OH_2_/Fe-OH equilibria, O-O cleavage, and site regeneration. Bulk pH is an operational descriptor, whereas the mechanistic variables are local proton activity, hydrogen-bond organization, and solvation around the Fe-oxidant complex (Figure 10c) [57]. Confined or highly functionalized microenvironments can sustain water and proton configurations distinct from the bulk phase and thereby change the relative free energies of radical cleavage, Fe-oxo formation, and interfacial electron transfer [49,57]. Models that neglect explicit or adequately represented solvation may therefore misidentify intermediate stability and rate-limiting steps (Figure 10d) [63]. Any perturbation that reorganizes hydration, proton availability, or competitive coordination can redistribute the working-state population and alter the dominant catalytic flux.

Collectively, pathway selection in Fe single-atom advanced oxidation systems cannot be predicted solely from catalyst structure or oxidant identity. Instead, it emerges from dynamic coupling among the Fe coordination environment, oxidant-bound working states, substrate electronic properties, and interfacial solvent structure. This ensemble perspective explains why identical Fe motifs can exhibit distinct oxidation pathways under different reaction environments and provides a more realistic framework for designing selective and durable oxidation catalysts.

### 3.4. Unified Dynamic Catalytic Cycle, Working-State Formation, Turnover, Regeneration, and Deactivation

The preceding pathways can be unified within a common kinetic architecture in which Fe single-atom catalysis proceeds through continuous transitions among coordination states. The resting ensemble first forms an oxidant-bound precursor, which partitions into pathway-specific reactive states. Substrate conversion is followed either by regeneration of the resting ensemble or by irreversible escape into a deactivated state. Radical, Fe-oxo, ^1^O_2_, and electron-transfer chemistry are therefore competing kinetic exits from a shared working-state network rather than independent mechanisms. Their contributions are governed by state formation, substrate reaction, coproduct release, and site regeneration rather than by the detectability of one intermediate. Catalytic performance ultimately reflects competition between productive turnover and irreversible deactivation. Operando studies show that structurally related Fe populations can differ markedly in their capacity for regeneration [28,64]. Nominal structural similarity therefore does not guarantee an equivalent catalytic trajectory. The design target is sustained flux through a reversible and regenerable coordination cycle, not maximum reactivity of an isolated intermediate.

This unified framework provides a mechanistic basis for understanding why structurally similar Fe single-atom catalysts can exhibit different oxidation behaviors under different reaction environments. Coordination microenvironments regulate not only the accessibility of reactive states but also their lifetime, utilization efficiency, and regeneration probability. Therefore, rational catalyst design should move beyond maximizing the formation of a specific oxidizing species and instead aim to program the entire dynamic catalytic trajectory from working-state generation to active-site recovery.

## 4. Engineering Dynamic Fe Single-Atom Microenvironments

### 4.1. Engineering Coordination Lability and Geometric Asymmetry for Working-State Regulation

#### 4.1.1. First-Shell Coordination-Number Regulation

**Figure 11:**
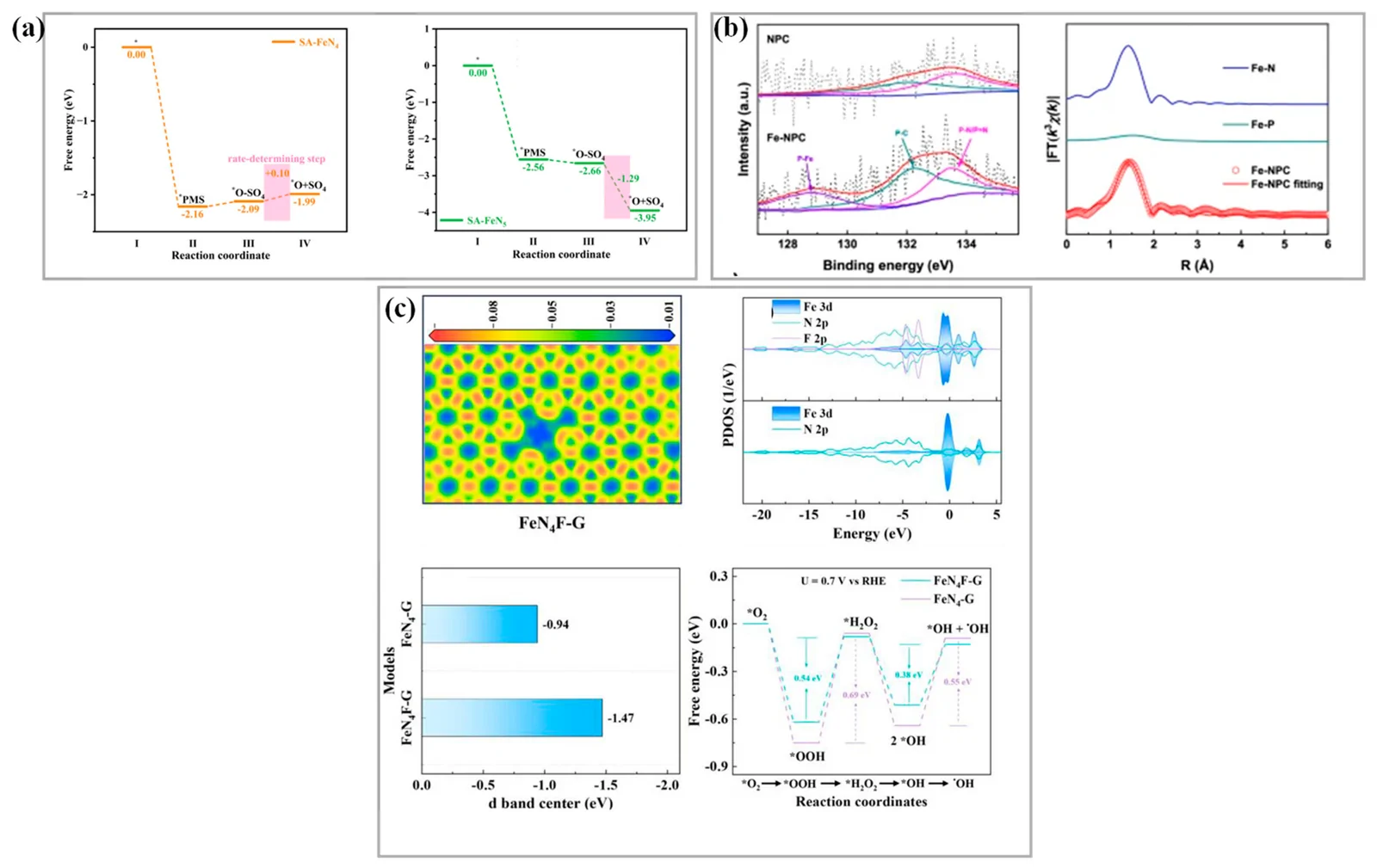
First-shell and axial coordination strategies relevant to Fe-site reactivity. (**a**) Coordination-dependent free-energy profiles for PMS activation at Fe-N_x_ sites; reproduced from Ref. [25]. (**b**) Fe-N_3_P_1_ first-shell asymmetry in catalytic ozonation, included as a cross-oxidant structural analogy; reproduced from Ref. [47]. (**c**) Axial-F coordination and its effects on oxygen activation in an electro-Fenton catalyst; reproduced from Ref. [54]. Reprinted with permission from Ref. [25] (Copyright 2024, Springer Nature), [47] (Copyright 2025, Springer Nature), and [54] (Copyright 2026, Wiley), respectively. The asterisk (*) denotes a surface site.

The design-relevant variable is not the nominal Fe-N coordination number itself but the energetic cost and reversibility of coordination opening during oxidant activation and site regeneration. A productive site must exchange solvent for peroxide, reach the required oxidant-bound geometry, release hydroxide or sulfate-derived ligands, and return to its anchoring environment without permanent Fe displacement [22,25]. Matched Fe-N_2_, Fe-N_3_, and Fe-N_4_ systems illustrate the reaction dependence. Lower N coordination favors Fe(IV)=O formation during PMS activation (Figure 11a), whereas Fe-N_4_ better supports Fe-oxo turnover in H_2_O_2_-driven benzene oxidation [22,65]. Coordination number therefore governs the accessibility, lifetime, and reversibility of catalytic trajectories rather than defining an intrinsic activity ranking.

This formulation avoids treating a dry-state vacancy as a persistent aqueous structure. Under turnover, solvation can reconstruct the nominally open coordination environment before oxidant activation. H_2_O or OH^−^ may occupy an open position before peroxide arrives. The design target is therefore a labile exchange site whose solvent ligand can be displaced during activation and restored during regeneration. The optimum first shell balances oxidant entry, intermediate release, and Fe retention over repeated turnover.

#### 4.1.2. Heteroatom-Induced First-Shell Asymmetry and Ligand-Field Regulation

Replacing an N ligand with a chemically distinct donor introduces asymmetry without necessarily creating additional coordination space. S, O, B, P, and C differ in donor strength, polarizability, electronegativity, orbital dimensions, and Fe-X covalency. Their incorporation can redistribute charge, split Fe d levels, alter spin-state accessibility, and impose directional oxidant binding. Because direct Fe-X bonding participates in the primary ligand field, it generally exerts a stronger local perturbation than remote substitution. The governing principle is symmetry breaking rather than heteroatom doping itself. Fe-N_3_P_1_ structures, for example, show that replacement of one N by P disrupts the charge symmetry of an Fe-N_4_-like environment and changes Fe-centered oxidation chemistry (Figure 11b) [47]. Although this evidence derives from catalytic ozonation rather than peroxide activation, it provides direct structural support for asymmetric first-shell engineering. Stronger perturbation, however, does not necessarily improve durability.

Evidence for S-, O-, and B-containing Fe environments indicates that these ligands can also bias peroxide chemistry, but the structural certainty and mechanistic maturity are uneven. In many pyrolyzed catalysts, distinguishing Fe-N_3_X from nearby nonbonded X using EXAFS alone remains challenging. A rigorous structure–mechanism assignment should therefore separate the chemical plausibility of heteroatom regulation, the structural evidence for Fe-X bonding, and the demonstrated catalytic consequence.

Excessive first-shell perturbation may accelerate working-state formation while simultaneously lowering the barrier for irreversible coordination reconstruction. Strong first-shell polarization can facilitate formation of an oxidant-bound state yet increase Fe displacement under axial pulling. Comparison with second-shell regulation shows that comparable electronic modulation can sometimes be achieved without weakening the primary Fe-N framework [16]. The design objective is thus not maximum charge separation but sufficient ligand-field anisotropy to bias the desired working state while retaining a recoverable Fe coordination geometry.

#### 4.1.3. Axial-Ligand Engineering

Axial coordination provides an especially direct means of programming the dynamic electronic structure and coordination response of Fe centers because the axial direction is also the principal trajectory through which water, peroxide, hydroxy, and oxo ligands interact with a planar Fe-N_4_ center. A preinstalled axial ligand converts an approximately two-dimensional ligand field into a three-dimensional coordination environment without removing the equatorial anchoring framework.

Axial O coordination provides a clear demonstration of three-dimensional ligand-field regulation without disrupting the equatorial Fe-N framework. In an FePc-O-nanodiamond configuration, the axial Fe-O interaction shifts Fe from a high-spin to an intermediate-spin state and reorganizes the 3dz^2^ orbital, thereby directing inner-sphere peroxide electron transfer [48]. Although this system activates peracetic acid rather than H_2_O_2_ or PMS, it establishes a transferable coordination principle in which axial ligation controls the orbital entrance to peroxide activation.

Axial coordination can reorganize Fe-centered electronic states and thereby influence the accessibility of peroxide-derived working states, but axial ligands must be distinguished by their persistence under reaction conditions. Studies of high-spin hydroxyl-ligated FeN_4_ in oxygen reduction [51] are relevant here as coordination analogies only; they do not demonstrate a hydroxylated Fe-SAC working state in Fenton-like oxidation. A more directly related example is axially F-coordinated FeN_4_F in electro-Fenton chemistry, where electronic redistribution facilitates the O_2_-to-H_2_O_2_-to-•OH sequence and improves tolerance to inorganic anions (Figure 11c) [54]. An axial ligand in an Fe-N_4_ catalyst can alter Fe Lewis acidity, spin, and opposite-side oxidant binding; the classical molecular trans effect should not be assumed without an appropriate structural comparison. Permanent axial ligands must retain the intended ligand field, whereas transient ligands must exchange on the timescale of peroxide binding, activation, product release, and site regeneration.

Collectively, first-shell coordination engineering should not aim to create a maximally activated Fe center, but rather to construct a coordination environment with optimized accessibility, electronic asymmetry, and structural recoverability. Coordination number, heteroatom identity, and axial ligation represent complementary strategies for regulating the formation and lifetime of Fe working states. Their optimal combination determines whether oxidant activation proceeds through selective and sustainable catalytic trajectories.

### 4.2. Engineering Extended and Cooperative Microenvironments Beyond the First Coordination Shell

First-shell modification is powerful but structurally costly. A complementary strategy is to regulate Fe through electronically and spatially coupled environments outside its direct coordination shell. Second-shell atoms, defects, neighboring sites, and confined solvent can tune Fe electronically or kinetically while leaving the primary Fe-N framework substantially intact. These factors define the boundary conditions under which oxidant-bound working states are formed, stabilized, and regenerated. The design unit therefore expands from an isolated Fe coordination polyhedron to an extended and dynamically responsive reaction field.

#### 4.2.1. Second-Shell Electronic Regulation and Long-Range Field Modulation

Second-shell regulation offers a route to decouple electronic activation from metal anchoring. This decoupling is particularly valuable because it enables enhancement of oxidant activation without sacrificing the structural integrity of the Fe-N framework. In Fe-N_4_-C_6_O_2_, remote O atoms amplify the local electric field and modify Fe electron density while preserving the Fe-N_4_ primary shell. This configuration suppresses excessive Fe-N deformation yet lowers the Fe=O acceptor-orbital energy and enhances Fe(IV)=O reactivity [16]. The mechanistic importance is not simply that “O doping improves activity,” but that electronic redistribution and structural confinement can be independently regulated across different length scales.

**Figure 12:**
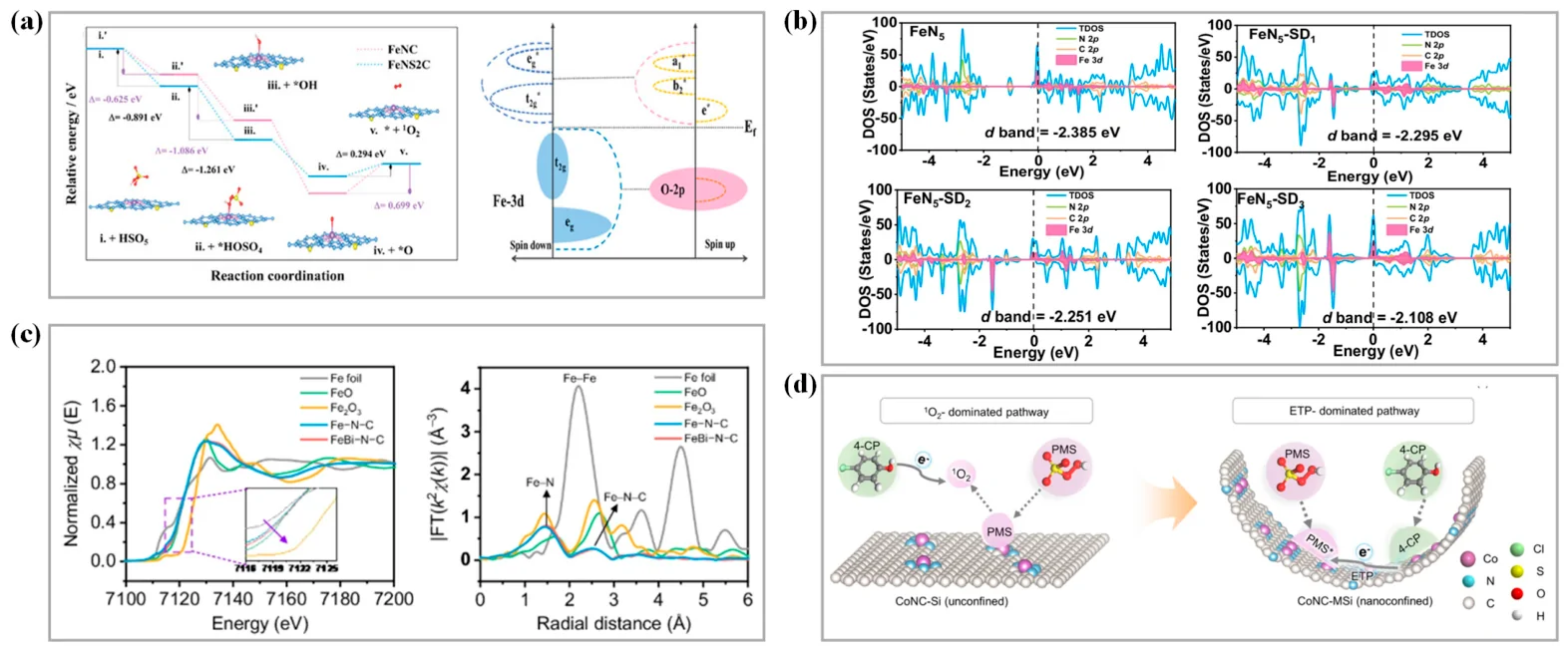
Extended and cooperative microenvironment engineering beyond the first Fe coordination shell. (**a**) Higher-shell S regulation of Fe electronic structure, PMS adsorption, and singlet-oxygen formation. The asterisk (*) denotes a surface site. Reprinted with permission from Ref. [66]. Copyright 2021, Wiley. (**b**) Defect-mediated remote tuning of Fe-N_5_ sites, showing a volcano relationship between defect density and PMS activation. Reprinted with permission from Ref. [31]. Copyright 2025, Springer Nature. (**c**) Atomically adjacent Fe-Bi sites forming a cooperative PMS-activation pocket that promotes Fe(IV)=O generation. Reprinted with permission from Ref. [34]. Copyright 2025, Springer Nature. (**d**) Nanoconfinement-induced oxidant enrichment and diffusion regulation, redirecting nonradical oxidation from ^1^O_2_ toward interfacial electron transfer. Reprinted with permission from Ref. [60]. Copyright 2024, Springer Nature.

Second-shell S and P regulation has also been proposed to perturb Fe d occupation and spin, although shell assignment is generally more model-dependent than for structurally constrained oxygen environments. Such claims require particular caution because a heteroatom detected near Fe may represent direct coordination, second-shell substitution, or statistical proximity. Misassignment of shell location can fundamentally alter the proposed structure–mechanism relationship. Shell-resolved XAS, matched first-/second-shell controls, and chemically credible structural models are therefore essential. Higher-shell S coordination further demonstrates that remote heteroatoms can regulate Fe spin populations, PMS adsorption, and pathway selectivity without replacing the primary Fe-N ligands (Figure 12a) [66].

Topological defects provide a long-range perturbation without introducing a new ligand. Vacancies and nonhexagonal carbon structures redistribute π charge and mechanical strain over several bonds. Long-range interactions between Fe-N_4_ centers can alter PMS activation without direct Fe-Fe bonding [30], whereas deliberately varied higher-shell defects around a well-defined Fe-N_5_ site generate a volcano relationship among defect density, PMS adsorption, and catalytic performance [31]. This nonmonotonic response shows that remote perturbation must be optimized rather than maximized (Figure 12b). Weak perturbation produces little electronic response, whereas excessive perturbation can overbind the oxidant, localize charge, impede transport, or weaken the support. The relevant design variables are therefore perturbation strength, spatial distribution, and decay length rather than total defect or heteroatom content.

#### 4.2.2. Neighboring-Site Cooperation and Cooperative Oxidant Activation

A neighboring atom can introduce a reaction coordinate unavailable to an isolated Fe center. One site may serve as the principal redox center, whereas another binds a second oxidant oxygen, accepts a proton, stabilizes a leaving group, or supplies charge. The value of a multisite microenvironment lies in this division of catalytic function. Fe-p-block ensembles provide a direct example. Bi, In, and Sb positioned near Fe interact preferentially with the hydroxyl oxygen of PMS while reorganizing Fe electron density and spin. This cooperative pocket shifts the reaction from an Fe-PMS*-mediated electron-transfer regime toward selective Fe(IV)=O generation [34]. Atomic-scale Fe-Bi proximity is shown in Figure 12c. The promoter does not simply make Fe more electron-rich. It changes oxidant geometry, polarization, and activation mode within the reaction pocket.

Fe-Fe cooperation can arise through a different route. An Fe-N-C-PMS* complex formed at one Fe center can interact with a suitably positioned neighboring Fe site, enabling additional charge transfer and Fe(IV)=O formation [32]. Intersite distance then becomes a mechanistic descriptor because electronic coupling and adsorbate bridging require an appropriate spatial relationship. Predefined atomic pairs should nevertheless be distinguished from reaction-induced cooperative ensembles. A dual-atom site exists before reaction, whereas a dynamic pair emerges only after an adsorbate or bridge creates electronic coupling. Operando studies in Fe-containing dual-site electrocatalysts show that M-O-Fe configurations can form under operation even when the resting structure differs [67]. Cooperative-site engineering must therefore balance electronic complementarity, distance, and stability. Excessive separation eliminates communication, whereas excessive proximity promotes aggregation. The optimum distance supports adsorbate-mediated coupling without sacrificing atomic isolation.

#### 4.2.3. Defect-Mediated Confinement and Interfacial Solvation Networks

The catalytic microenvironment is defined by coupled electronic, spatial, and solvation fields. Pore geometry controls the transport of oxidant, pollutant, water, ions, and products to and from the Fe center. The same Fe-N_4_ motif can therefore experience different reaction fields in an open mesopore, a narrow micropore, or a polar molecular cavity. Confinement can enrich reactants and shorten the distance between intermediate generation and substrate attack, but its benefit depends on whether residence time promotes turnover rather than accumulation. Nanoconfined Fenton-like systems show that restricted volumes can shift the balance between diffusional radical chemistry and surface-associated nonradical oxidation [68]. Restricted diffusion can also redirect the dominant nonradical pathway (Figure 12d) [60], whereas co-enrichment of oxidant and pollutant increases the probability that an activated state is consumed productively [69]. The relevant descriptor is productive residence time coupled to exchange kinetics. A pore must retain oxidant and substrate long enough for reaction while allowing hydroxide, sulfate, partially oxidized products, and water to exchange rapidly enough for regeneration. Excessive confinement converts enrichment into poisoning.

Local wettability governs another component of this balance. Hydrophilic environments facilitate water and ionic-oxidant access but can form structured hydration shells that compete for axial coordination. Hydrophobic regions enrich aromatic substrates but may restrict aqueous oxidant delivery. Macroscopic contact angles are therefore insufficient descriptors. The mechanistically relevant quantity is the solvation structure within molecular distance of Fe. Explicit-water calculations show that water population and orientation respond to the electronic state of Fe-N_4_ interfaces and modify reaction energetics [13]. Hydrogen-bond networks can relay protons among peroxide, Fe-OH, neighboring N/O groups, and bulk water. Defects and heteroatoms therefore regulate catalysis through coupled electronic and solvation effects. An optimized microenvironment should provide a hydrated pathway for oxidant and proton delivery, an adsorption domain for pollutant enrichment, and an open route for product and coproduct removal. This organization is the heterogeneous analogue of a reaction pocket.

Extended-microenvironment engineering expands catalyst design beyond direct Fe coordination. Second-shell atoms, neighboring sites, defects, confinement, and interfacial solvent networks regulate the electronic field, spatial organization, and dynamic accessibility of Fe working states. Effective microenvironments do not maximize one interaction. They coordinate oxidant activation, intermediate stabilization, substrate conversion, coproduct release, and site regeneration.

### 4.3. Framework-Confined Platforms for Programming Dynamic Fe Coordination Microenvironments

#### 4.3.1. Molecularly Defined Fe Coordination Pockets

**Figure 13:**
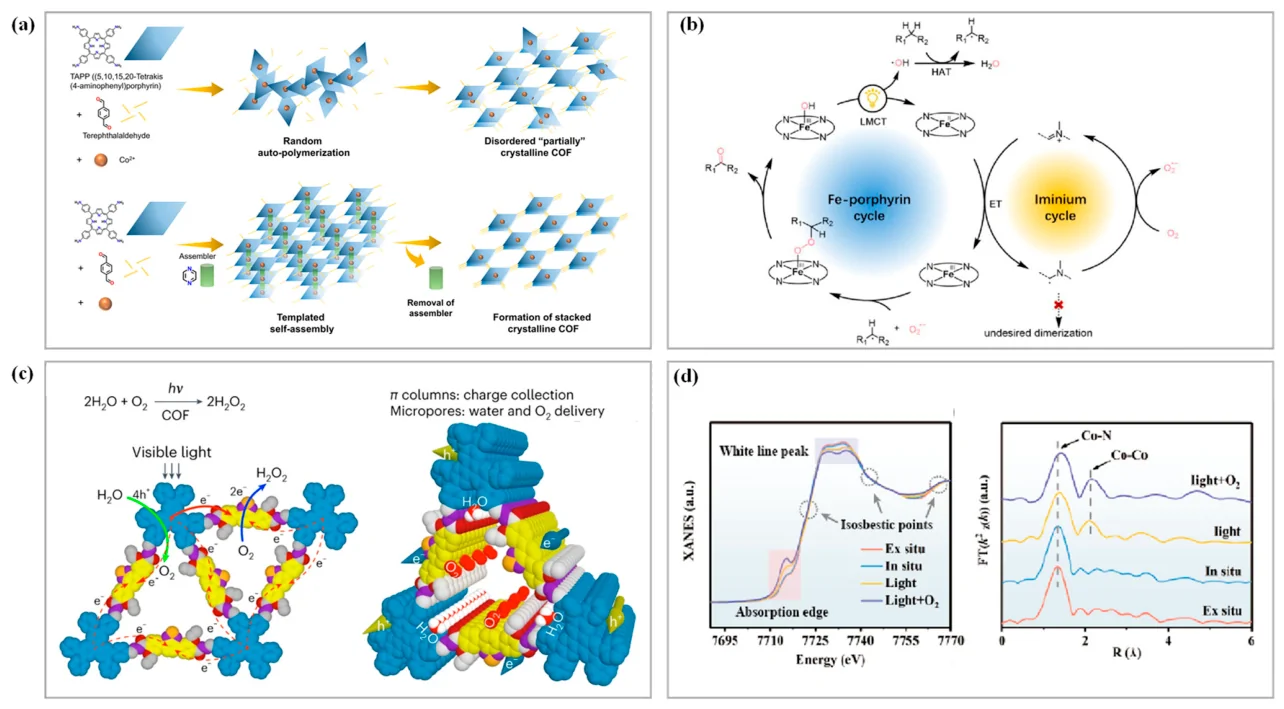
Framework-confined regulation of dynamic metal coordination microenvironments. (**a**) Standard and assembler-assisted synthesis of crystalline metalloporphyrin COFs with molecularly defined M-N_4_ pockets. Reprinted with permission from Ref. [44]. Copyright 2024, American Chemical Society. (**b**) Cooperative Fe-porphyrin ligand-to-metal charge transfer and linkage-mediated electron transfer during O_2_ activation. Reprinted with permission from Ref. [45]. Copyright 2026, American Chemical Society. (**c**) A donor-acceptor COF integrating charge-transporting π columns with oxygen-lined channels for water and O_2_ delivery. Reprinted with permission from Ref. [40]. Copyright 2024, Springer Nature. (**d**) Operando evolution of isolated Co atoms into transient atom pairs in a pyridyl CTF during photocatalytic H_2_O_2_ production. Reprinted with permission from Ref. [39]. Copyright 2024, American Chemical Society. The asterisk (*) denotes a surface site.

Pyridyl, bipyridyl, phenanthroline, porphyrin, phthalocyanine, and triazine units provide distinct approaches to Fe confinement. Their key value lies not only in metal capture but also in defining the initial coordination landscape from which catalytic states emerge. Chelating N linkers prescribe donor separation and geometry, whereas porphyrinic N_4_ cavities provide strongly preorganized pockets. Triazine-rich frameworks offer more distributed N coordination embedded within an extended π system. Metal incorporation can be performed through premetalated building blocks or postsynthetic metalation. Premetalation preserves a predefined coordination pocket during framework formation; postsynthetic metalation permits metal loading and site occupancy to be adjusted after crystallization. This distinction is mechanistically important because premetalated and postsynthetically metalated sites may exhibit different distributions of local coordination environments [36]. Premetalation and assembler-assisted synthesis provide complementary routes for retaining molecularly defined metalloporphyrin pockets within crystalline frameworks (Figure 13a) [44].

The distinction from pyrolyzed COF-derived Fe-N-C is essential. Carbonization reconstructs the original covalent network and generates new Fe-N/C environments; catalytic behavior of the resulting carbon cannot be used as direct evidence for the coordination chemistry of the intact parent COF. Molecular programmability is retained only when the structural relationship between linker and Fe survives the catalytic material preparation and remains relevant under reaction conditions. Fe loading should also be interpreted through nuclearity rather than mass percentage alone. A framework that precisely controls Fe spacing can intentionally access cooperative dual-site chemistry or prevent undesired aggregation. Increasing site density is useful only while the intended pockets remain chemically distinguishable. Once Fe-Fe interactions become appreciable, the system may deliberately enter cooperative-site chemistry or unintentionally nucleate clusters. Reticular chemistry is valuable precisely because these possibilities can be designed through linker topology and site spacing.

#### 4.3.2. Framework-Fe Electronic Coupling for Working-State Regulation

A framework-bound Fe center is electronically coupled to the COF and should be treated as part of an extended ligand environment rather than as a supported molecular fragment. The π skeleton controls how excitation and charge migrate to or from the metal pocket. Porphyrinic COFs show that metalation can alter exciton dissociation and redirect O_2_ activation without changing framework topology [70]. Bifunctional Fe-porphyrin frameworks provide a more direct example. Ligand-to-metal charge transfer at Fe and electron transfer through adjacent redox-active linkages operate cooperatively during O_2_ activation (Figure 13b) [45]. The framework therefore participates in the Fe redox sequence instead of acting as an inert porous scaffold.

Donor–acceptor architectures offer a means of controlling this coupling. Their function is not merely to narrow the band gap but to determine where charges separate, how rapidly they migrate, and whether the Fe pocket acts as an electron sink, redox relay, or terminal catalytic center. Insufficient framework-metal coupling isolates Fe from photogenerated charge, whereas excessive localization at Fe can accelerate nonproductive recombination. The design target is directional electronic coupling on the timescale required for working-state formation. Linker chemistry can also modify Fe valence and spin through conjugation, local electric fields, and axial interactions. Framework–metal coupling is therefore bidirectional. The framework tunes Fe electronic structure, and Fe incorporation reorganizes local charge distribution and excited-state dynamics within the framework.

#### 4.3.3. Pore-Confined Solvation and Transport Regulation of Fe Working States

COF pores provide an unusual opportunity to couple molecular coordination with controlled solvation. Pore aperture and layer stacking regulate diffusion of O_2_, H_2_O_2_, PMS/PDS, water, and organic pollutants, whereas pore-wall functionality governs polarity and hydrogen bonding. Studies of COF-based H_2_O_2_ photosynthesis provide transferable design principles, although their application to Fe-mediated oxidation requires direct validation. Linkage-engineered donor–acceptor COFs combine charge separation with controlled transport [40], and hierarchical pore-skeleton design can coordinate water and air delivery to photocatalytic domains [41]. Oxygen-lined one-dimensional channels illustrate how linkage engineering integrates framework charge transport with directional water and O_2_ delivery (Figure 13c) [40]. The dry crystallographic pore does not represent the catalytic microenvironment under aqueous turnover. Protonated linkers, hydration layers, counterions, pollutants, and oxidants form a dynamic pore phase, while pyridyl, triazine, imine, and carbonyl groups organize hydrogen-bond networks that can influence proton delivery to Fe-peroxide and Fe-OH states.

Framework flexibility adds a further variable. Moderate compliance may accommodate reversible Fe displacement or ligand exchange, whereas excessive swelling can erase geometric definition and excessive rigidity can raise the energetic cost of working-state formation. Direct Fe-specific operando evidence linking COF breathing to Fe working-state geometry remains scarce. Framework flexibility should therefore be presented as a testable design hypothesis rather than an established universal advantage.

#### 4.3.4. Coupled Oxidant Generation and Fe Working-State Activation

Photocatalytic COFs offer the possibility of integrating oxidant production with Fe-mediated activation. Donor–acceptor COFs can generate H_2_O_2_ directly from O_2_ and water, and linkage and pore engineering have substantially improved this chemistry [40,41,42]. Crystalline CTFs can likewise couple two-electron oxygen reduction with water oxidation to produce H_2_O_2_ [43]. Embedding an Fe pocket creates a proposed sequence in which photon absorption and charge separation generate H_2_O_2_, followed by Fe capture, working-state formation, pollutant oxidation, and Fe regeneration. The central requirement is kinetic matching rather than maximum H_2_O_2_ production. Excess production allows peroxide to accumulate and escape the reaction pocket, whereas insufficient production underuses the Fe population. Productive integration requires comparable rates of charge generation, O_2_ reduction, H_2_O_2_ transport, Fe-peroxide formation, and Fe-site regeneration.

The evidence for this integrated sequence must be graded carefully. COFs are established H_2_O_2_ photocatalysts, and Fe-containing COFs demonstrate framework–Fe electronic coupling. Direct operando observation of H_2_O_2_ formation, Fe-OOH generation, Fe-oxo or radical production, and Fe regeneration within an intact Fe-COF remains limited. The concept should therefore be presented as a major design opportunity rather than a closed mechanism. The same caution applies to light-assisted PMS/PDS activation. Increased oxidant consumption alone does not establish productive coupling. The decisive evidence is whether photogenerated charge changes the population and regenerative flux of the intended Fe working state.

CTFs provide a focused example rather than a separate materials branch. Triazine-rich coordination, conjugated transport, and framework rigidity can support H_2_O_2_ photosynthesis [43]. Operando work on Co/CTF further shows that a framework-confined single atom can adopt a working nuclearity different from its resting nuclearity (Figure 13d) [39]. This Co precedent should not be extrapolated directly to Fe. It establishes the need for Fe-specific measurements that follow coordination from light absorption and peroxide generation through activation and site recovery. COFs already demonstrate control over binding geometry, electronic coupling, pore transport, and oxidant generation, but closed-loop control of the Fe working-state population remains unproven. Claims of programmable Fe microenvironments should therefore distinguish structurally encoded framework variables from directly observed working-state responses.

Framework-confined platforms provide a distinct opportunity to translate coordination chemistry into programmable catalytic architectures. COFs and related porous frameworks can encode Fe-binding geometry, electronic coupling, spatial confinement, and oxidant-delivery pathways. Structural programmability, however, should not be equated with direct control of catalytic working states. Progress will depend on operando validation that connects encoded framework features with the evolution of Fe coordination, oxidant activation, substrate conversion, and site regeneration.

### 4.4. Environmental Constraints on Working-State Stability and Catalytic Trajectory Preservation

A deliberately engineered working-state landscape remains catalytically meaningful only if it persists under realistic chemical and operational perturbations. Matrix ligands, pH fluctuations, natural organic matter, framework oxidation, pore fouling, and cumulative oxidant exposure can alter both the distribution of accessible Fe states and the population of sites capable of sustaining turnover. Three distinct but interconnected levels of stability should therefore be considered. Structural stability concerns retention of the Fe site and supporting framework, working-state stability concerns preservation of the reaction-induced Fe working-state population and its dynamic accessibility, and functional stability concerns whether catalytic flux continues to follow the intended pathway. A catalyst may retain an apparent Fe-N_4_ structure while the population of Fe(IV)=O, the contribution of interfacial electron transfer, or the kinetics of site regeneration change substantially. Environmental robustness should therefore be defined by preservation of productive and regenerable catalytic trajectories, rather than by catalyst retention or short-term pollutant removal alone.

#### 4.4.1. Competitive Coordination and Interfacial Water-Matrix Regulation

**Figure 14:**
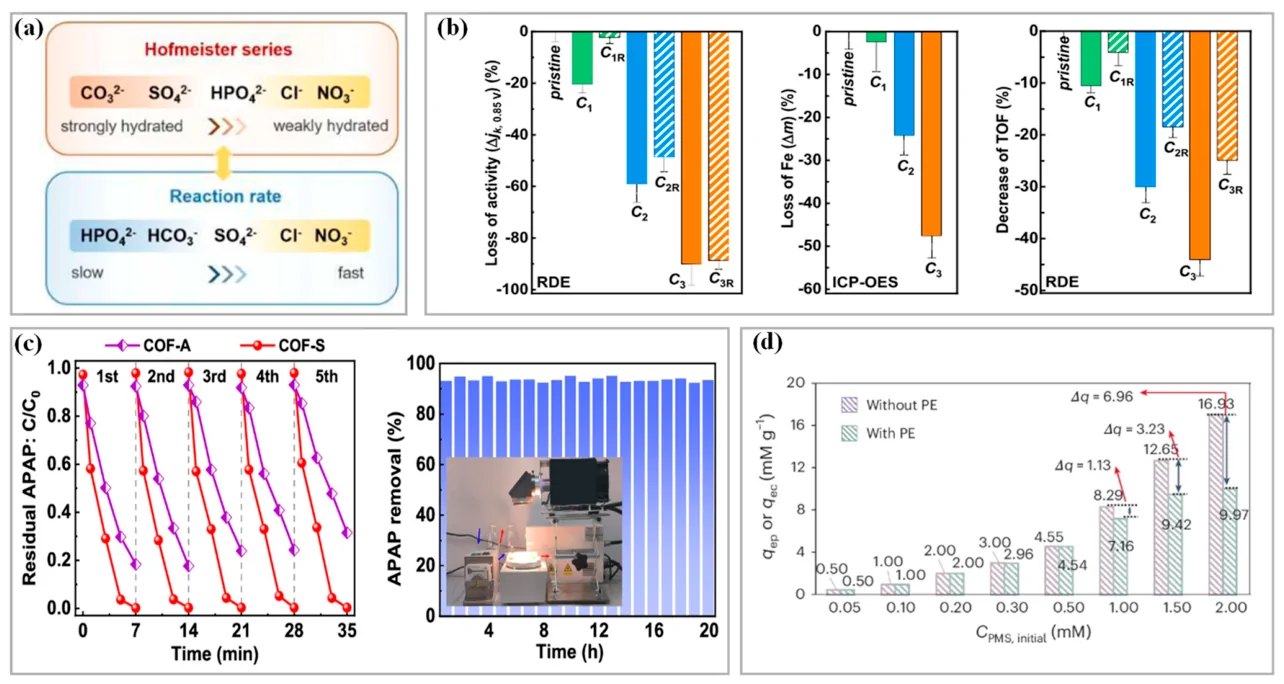
Environmental constraints governing working-state stability and catalytic-trajectory preservation. (**a**) Anion-dependent perturbation of heterogeneous persulfate oxidation associated with ion hydration and interfacial interactions. Reprinted with permission from Ref. [71]. Copyright 2025, American Chemical Society. (**b**) Decoupled losses of catalytic activity, Fe-site inventory, and turnover frequency in Fe-N-C under acidic, O_2_, and ROS stress. Reprinted with permission from Ref. [73]. Copyright 2025, American Chemical Society. (**c**) Improved cycling and continuous-flow water purification enabled by rigid thiazole-linked COFs. Reprinted with permission from Ref. [74]. Copyright 2024, Springer Nature. (**d**) Polymeric-product deposition suppressing oxidant adsorption and interfacial electron transfer on carbon catalysts. Reprinted with permission from Ref. [75]. Copyright 2025, Springer Nature.

Common anions affect heterogeneous AOPs through more than radical scavenging; Cl^−^, HCO_3_^−^, phosphate, sulfate, and nitrate can alter electrostatics, oxidant adsorption, interfacial bonding, proton activity, and hydration before reactive oxygen species are generated [71]. Dual-substrate analysis links these effects to ion-specific hydration and interfacial interactions (Figure 14a) [71]. Competitive ligation is particularly important for Fe SACs. Phosphate can occupy strongly polarized interfacial sites, hydroxide changes axial Fe speciation, bicarbonate couples radical chemistry with acid-base equilibria, and chloride can enter secondary oxidation chemistry. The consequences depend on the dominant working state. Diffusible-radical pathways are especially sensitive to bulk interception, whereas Fe-oxo and electron-transfer pathways can be more sensitive to coordination competition and substrate adsorption.

NOM introduces additional complexity. It can intercept radicals, absorb photons, occupy pore surfaces, donate electrons to activated oxidants, and alter transformation-product chemistry [72]. On porous Fe/COFs, high-molecular-weight fractions may also restrict access to internal pockets. “NOM tolerance” is therefore meaningful only when oxidant consumption, pollutant uptake, and accessible Fe-site density are assessed together. pH couples all of these processes through Fe-OH_2_/Fe-OH speciation, pollutant ionization, peroxide protonation, framework charge, and proton-transfer kinetics. Bulk pH is an operating variable; the mechanistic variable is the local proton and ligand environment experienced by Fe.

#### 4.4.2. Framework Degradation, Fe Escape, and Accessibility Loss

Catalytic flexibility becomes degradation when the Fe site no longer returns to its anchoring environment. This transition from reversible adaptation to irreversible escape defines the mechanistic boundary of failure. Fe-N-C studies show that distinct FeNx populations possess different intrinsic durability and that active-site loss can evolve independently of the turnover frequency of surviving sites [28,64,73]. Quantitative separation of activity decay, Fe-site loss, and intrinsic TOF decline therefore provides a more informative diagnosis than bulk activity alone (Figure 14b) [73].

In intact Fe/COFs, framework linkage introduces an additional failure coordinate. Oxidation or hydrolysis of the covalent skeleton can weaken the molecular pocket before measurable Fe loss occurs. Converting dynamic imine bonds into rigid thiazole linkages improves framework retention and continuous photocatalytic water purification (Figure 14c) [74]. Framework degradation and Fe leaching should therefore be quantified separately. ICP analysis reports metal release but not the integrity of the organic pocket, whereas PXRD may preserve an average lattice after selective loss of catalytically important sites. Organic-carbon release and linker fragments are needed to identify framework dissolution.

Pore fouling is a third and mechanistically distinct route to deactivation. Oxidation products, natural organic matter, inorganic precipitates, and polymeric species can block access to intact Fe sites without destroying their coordination structure. Polymeric oxidation products have been shown to adhere to carbon surfaces, suppress oxidant adsorption and interfacial electron transfer, and deactivate otherwise intact catalytic sites (Figure 14d) [75]. This accessibility loss is particularly important for COFs because pore architecture is deliberately coupled to active-site access. A complete stability diagnosis should therefore resolve Fe-site retention, framework integrity, pore accessibility, and Fe nuclearity independently.

#### 4.4.3. Continuous-Flow Lifetime Evaluation and Working-State-Based Performance Metrics

Repeated batch cycles are a weak proxy for operational durability because washing and redosing remove accumulated products and repeatedly reset mass-transfer conditions. Continuous-flow operation is more informative because oxidant exposure, fouling, matrix competition, and product accumulation remain coupled over time. COF water-treatment systems have already demonstrated that framework and pore design can be translated into continuous or membrane operation [42,74]. Lifetime should be normalized not only to operating hours but also to cumulative pollutant loading, treated-water volume, and oxidant exposure. Flow rate, hydraulic residence time, oxidant concentration, pH, ionic strength, and NOM loading must accompany lifetime claims. Parent-pollutant disappearance is likewise insufficient. A catalyst that rapidly consumes oxidants through nonproductive pathways should not be considered highly efficient. At minimum, practical assessment should integrate mineralization, oxidant utilization, transformation-product toxicity, Fe and organic-framework leaching, and energy or chemical input. Fast disappearance accompanied by low mineralization or nonproductive peroxide decomposition is not equivalent to efficient treatment.

Intrinsic catalytic stability requires normalization to active sites. Mass-normalized pseudo-first-order rates cannot distinguish loss of Fe-site population from loss of turnover frequency at surviving sites, and these failure modes require different optimization strategies. Site density, TOF, and cumulative turnover number should therefore be followed whenever experimentally accessible [64]. The most rigorous lifetime assessment closes both structural and kinetic balances across the experiment. It connects the fresh resting structure with the operando working-state population, continuous catalytic performance, and the post-operation site inventory. Operando Fe-sensitive spectroscopy should establish whether the intended working states remain accessible. ICP and organic-leaching analyses quantify irreversible structural escape, porosity measurements diagnose fouling, and post-operation spectroscopy and microscopy determine whether Fe retains its intended nuclearity and coordination environment.

Environmental stability should be evaluated as preservation of a dynamic catalytic trajectory rather than retention of the fresh catalyst structure alone. Water-matrix components, framework degradation, transport limitations, and cumulative oxidant exposure continuously reshape the Fe working-state population. Catalyst design should therefore integrate activity, selectivity, and durability by controlling both the generation and regeneration of reactive Fe states under realistic operating conditions.

## 5. Conclusions

Fe single-atom catalysis in advanced oxidation is governed by the coupling of resting coordination, the extended microenvironment, oxidant identity, pollutant chemistry, and reaction-induced working states. The framework advanced here connects structure, working state, pathway, and lifetime. Radical, Fe-oxo, singlet-oxygen, and interfacial electron-transfer pathways can compete within a shared coordination landscape; catalytic selectivity and durability therefore depend on working-state formation, consumption, and regeneration. Direct operando identification remains strongest for selected Fe-oxo systems, whereas a complete Fe-COF turnover cycle has not been established.

The first priority is quantitative operando identification. Future studies should resolve the concentration, lifetime, and interconversion kinetics of peroxide-bound, hydroxo, oxo, and electronically coupled Fe-oxidant states under turnover. Recent time-resolved Fe-oxo assignments illustrate the value of coupling spectral evolution to chemical conversion, while also exposing the need to establish kinetic competence for each assigned intermediate. Time-resolved XAS, Mössbauer spectroscopy, vibrational spectroscopy, isotope labeling, and kinetic perturbation should therefore be integrated within the same reaction window. These measurements are also needed to distinguish reversible coordination adaptation from irreversible demetallation, aggregation, and support oxidation.

The second priority is predictive modeling of the complete catalytic trajectory. Static adsorption energies on dry structural models cannot capture solvent exchange, proton relays, spin crossover, coproduct release, or site regeneration. Recent studies of higher-shell interactions, neighboring Fe sites, and proton-coupled interfacial activation show why the environment surrounding the first shell must be included in mechanistic models. Explicit-water simulations, free-energy sampling, and microkinetic models should be connected to experimentally measured working-state populations. Useful descriptors should predict sustained catalytic flux across the full cycle rather than the stability of one intermediate alone.

The third priority is to translate coordination control into realistic treatment architectures. First-shell asymmetry, axial ligation, second-shell fields, neighboring-site cooperation, and framework confinement should be optimized together with oxidant delivery and product removal. Recent work on framework transport and water-matrix effects provides practical constraints, but direct evidence for closed-loop control of Fe working states in COFs remains limited. Continuous-flow evaluation in the presence of background ions and natural organic matter should quantify active-site density, intrinsic turnover frequency, oxidant utilization, mineralization, transformation-product toxicity, Fe and framework leaching, pore accessibility, and cumulative turnover. Product-mediated catalyst fouling offers an additional reason to measure accessibility and working-state persistence over extended operation.

## Data Availability

No new data were created or analyzed in this study. Data sharing is not applicable to this article.

## References

[1] Miklos D.B., Remy C., Jekel M., Linden K.G., Drewes J.E., Hübner U. (2018). Evaluation of advanced oxidation processes for water and wastewater treatment—A critical review. Water Res..

[2] Lee J., Von Gunten U., Kim J. (2020). Persulfate-Based Advanced Oxidation, Critical Assessment of Opportunities and Roadblocks. Environ. Sci. Technol..

[3] Pignatello J.J., Oliveros E., MacKay A. (2006). Advanced oxidation processes for organic contaminant destruction based on the fenton reaction and related chemistry. Crit. Rev. Environ. Sci. Technol..

[4] Yan Y., Wei Z., Duan X., Long M., Spinney R., Dionysiou D.D., Xiao R., Alvarez P.J.J. (2023). Merits and Limitations of Radical vs. Nonradical Pathways in Persulfate-Based Advanced Oxidation Processes. Environ. Sci. Technol..

[5] Brillas E., Sire I., Oturan M.A. (2009). Electro-Fenton Process and Related Electrochemical Technologies Based on Fenton’s Reaction Chemistry. Chem. Rev..

[6] Yang L., Jiao Y., Xu X., Pan Y., Su C., Duan X., Sun H., Liu S., Wang S., Shao Z. (2022). Superstructures with Atomic-Level Arranged Perovskite and Oxide Layers for Advanced Oxidation with an Enhanced Non-Free Radical Pathway. ACS Sustain. Chem. Eng..

[7] Shang Y., Xu X., Gao B., Wang S., Duan X. (2021). Single-atom catalysis in advanced oxidation processes for environmental remediation. Chem. Soc. Rev..

[8] Meyerstein D. (2021). Re-examining Fenton and Fenton-like reactions. Nat. Rev. Chem..

[9] Lei Y., Yu Y., Lei X., Liang X., Cheng S., Ouyang G., Yang X. (2023). Assessing the Use of Probes and Quenchers for Understanding the Reactive Species in Advanced Oxidation Processes. Environ. Sci. Technol..

[10] Chen Y., Miller C.J., Xie J., Waite T.D. (2023). Challenges Relating to the Quantification of Ferryl(IV) Ion and Hydroxyl Radical Generation Rates Using Methyl Phenyl Sulfoxide (PMSO), Phthalhydrazide, and Benzoic Acid as Probe Compounds in the Homogeneous Fenton Reaction. Environ. Sci. Technol..

[11] Zong Y., Chen L., Wu J.-H., Liu F., Guan X., Lei Z., Xu J., Wu D., Liu W., Yu H.-Q. (2025). Pitfalls in Probing Singlet Oxygen by Electron Paramagnetic Resonance Spectroscopy in Engineered Environmental Systems. Environ. Sci. Technol..

[12] Guo J., Wang Y., Shang Y., Yin K., Li Q., Gao B., Li Y., Duan X., Xu X. (2024). Fenton-like activity and pathway modulation via single-atom sites and pollutants comediates the electron transfer process. Proc. Natl. Acad. Sci. USA.

[13] Wang Y., Yan H.-M., Cao H., Chen J., Yang H., Zhu J., Sun J. (2023). Atomic-Scale Understanding of Electrified Interfacial Structures and Dynamics during the Oxygen Reduction Reaction on the Fe-N_4_/C Electrocatalyst. ACS Catal..

[14] Yu S., Levell Z., Jiang Z., Zhao X., Liu Y. (2023). What Is the Rate-Limiting Step of Oxygen Reduction Reaction on Fe-N-C Catalysts?. J. Am. Chem. Soc..

[15] Zhao Q., Huang Q., Duan R., Zhang Z., Xie Y., Song W., Sheng H., Zhao J. (2025). Dynamic identification of reactive iron-oxo species in heterogeneous Fenton-like reaction via operando stopped-flow IR spectroscopy. Nat. Commun..

[16] Chen T., Zhang G., Sun H., Hua Y., Yang S., Zhou D., Di H., Xiong Y., Hou S., Xu H. (2025). Robust Fe-N_4_-C_6_O_2_ single-atom sites for efficient PMS activation and enhanced Fe(IV)=O reactivity. Nat. Commun..

[17] Wang A., Li J., Zhang T. (2018). Heterogeneous single-atom catalysis. Nat. Rev. Chem..

[18] Cui X., Li W., Ryabchuk P., Junge K., Beller M. (2018). Bridging homogeneous and heterogeneous catalysis by heterogeneous single-metal-site catalysts. Nat. Catal..

[19] Mitchell S., Pérez-Ramírez J. (2020). Single atom catalysis, a decade of stunning progress and the promise for a bright future. Nat. Commun..

[20] Wu X., Zhang H., Zuo S., Dong J., Li Y., Zhang J., Han Y. (2021). Engineering the Coordination Sphere of Isolated Active Sites to Explore the Intrinsic Activity in Single-Atom Catalysts. Nano-Micro Lett..

[21] Zitolo A., Goellner V., Armel V., Sougrati M.-T., Mineva T., Stievano L., Fonda E., Jaouen F. (2015). Identification of catalytic sites for oxygen reduction in iron- and nitrogen-doped graphene materials. Nat. Mater..

[22] Pan Y., Chen Y., Wu K., Chen Z., Liu S., Cao X., Cheong W.-C., Meng T., Luo J., Zheng L. (2019). Regulating the coordination structure of single-atom Fe-NxCy catalytic sites for benzene oxidation. Nat. Commun..

[23] Zhang L.-S., Jiang X.-H., Zhong Z.-A., Tian L., Sun Q., Cui Y.-T., Lu X., Zou J.-P., Luo S.-L. (2021). Carbon Nitride Supported High-Loading Fe Single-Atom Catalyst for Activation of Peroxymonosulfate to Generate ^1^O_2_ with 100% Selectivity. Angew. Chem. Int. Ed..

[24] Xiong Y., Li H., Liu C., Zheng L., Liu C., Wang J.-O., Liu S., Han Y., Gu L., Qian J. (2022). Single-Atom Fe Catalysts for Fenton-Like Reactions, Roles of Different N Species. Adv. Mater..

[25] Lin Y., Wang Y., Weng Z., Zhou Y., Liu S., Ou X., Xu X., Cai Y., Jiang J., Han B. (2024). Coordination engineering of heterogeneous high-valent Fe(IV)-oxo for safe removal of pollutants via powerful Fenton-like reactions. Nat. Commun..

[26] Sarma B.B., Maurer F., Doronkin D.E., Grunwaldt J.-D. (2022). Design of Single-Atom Catalysts and Tracking Their Fate Using Operando and Advanced X-ray Spectroscopic Tools. Chem. Rev..

[27] Ni L., Gallenkamp C., Wagner S., Bill E., Krewald V., Kramm U.I. (2022). Identification of the Catalytically Dominant Iron Environment in Iron- and Nitrogen-Doped Carbon Catalysts for the Oxygen Reduction Reaction. J. Am. Chem. Soc..

[28] Li J., Sougrati M.T., Zitolo A., Ablett J.M., Oğuz I.C., Mineva T., Matanovic I., Atanassov P., Huang Y., Zenyuk I. (2021). Identification of durable and non-durable FeNx sites in Fe-N-C materials for proton exchange membrane fuel cells. Nat. Catal..

[29] Chen Z., Jiang S., Kang G., Nguyen D., Schatz G.C., Van Duyne R.P. (2019). Operando Characterization of Iron Phthalocyanine Deactivation during Oxygen Reduction Reaction Using Electrochemical Tip-Enhanced Raman Spectroscopy. J. Am. Chem. Soc..

[30] Wu Z., Xiong Z., Huang B., Yao G., Zhan S., Lai B. (2024). Long-range interactions driving neighboring Fe-N_4_ sites in Fenton-like reactions for sustainable water decontamination. Nat. Commun..

[31] Jin S., Tan W., Huang Y., Wang Y., He Z., Zhang H., Song S., Cai Y., Zeng T. (2025). Remote tuning of single-atom Fe-N_5_ sites via high-coordination defects for enhanced Fenton-like water decontamination. Nat. Commun..

[32] Cheng C., Ren W., Miao F., Chen X., Chen X., Zhang H. (2023). Generation of FeIV= O and its Contribution to Fenton-Like Reactions on a Single-Atom Iron-N-C Catalyst. Angew. Chem. Int. Ed..

[33] Wei S., Sun Y., Qiu Y.-Z., Li A., Chiang C.-Y., Xiao H., Qian J., Li Y. (2023). Self-carbon-thermal-reduction strategy for boosting the Fenton-like activity of single Fe-N_4_ sites by carbon-defect engineering. Nat. Commun..

[34] Zhao Z., Dai H., Nie T., Hu T., Zhou W., Zhang M., Xu J., Lin D., Zhu L. (2025). Dynamic electronic modulation of single-atom Fe sites with p-block metal coordination enables highly selective generation of FeIV = O in Fenton-like reactions. Nat. Commun..

[35] Xu S., Draksharapu A., Rasheed W., Que L. (2019). Acid pKa Dependence in O-O Bond Heterolysis of a Nonheme FeIII-OOH Intermediate To Form a Potent FeV=O Oxidant with Heme Compound I-Like Reactivity. J. Am. Chem. Soc..

[36] Ran L., Li Z., Ran B., Cao J., Zhao Y., Shao T., Song Y., Leung M.K.H., Sun L., Hou J. (2022). Engineering Single-Atom Active Sites on Covalent Organic Frameworks for Boosting CO_2_ Photoreduction. J. Am. Chem. Soc..

[37] Ai L., Wang Q., Cui F., Jiang G. (2023). Peroxymonosulfate-Assisted Phenol Degradation via a Magnetic Covalent-Triazine-Framework-Based Photocatalyst. ChemCatChem.

[38] Diercks C.S., Yaghi O.M. (2017). The atom; the molecule, and the covalent organic framework. Science.

[39] Zhu C., Yao Y., Fang Q., Song S., Chen B., Shen Y. (2024). Unveiling the Dynamic Evolution of Single-Atom Co Sites in Covalent Triazine Frameworks for Enhanced H_2_O_2_ Photosynthesis. ACS Catal..

[40] Liu R., Chen Y., Yu H., Položij M., Guo Y., Sum T.C., Heine T., Jiang D. (2024). Linkage-engineered donor-acceptor covalent organic frameworks for optimal photosynthesis of hydrogen peroxide from water and air. Nat. Catal..

[41] Chen Y., Liu R., Guo Y., Wu G., Sum T.C., Yang S.W., Jiang D. (2024). Hierarchical assembly of donor-acceptor covalent organic frameworks for photosynthesis of hydrogen peroxide from water and air. Nat. Synth..

[42] Chen Y., Guo Y., Wang T., Ji S., Shao H., Lin M., Seki S., Yan N., Jiang D. (2025). Covalent organic framework photocatalysts for green and efficient photochemical transformations. Nat. Commun..

[43] Zhang L., Wang C., Jiang Q., Lyu P., Xu Y. (2024). Structurally Locked High-Crystalline Covalent Triazine Frameworks Enable Remarkable Overall Photosynthesis of Hydrogen Peroxide. J. Am. Chem. Soc..

[44] Bourda L., Dansette I., Jena H.S., Chen H., Raza A., Vrielinck H., Van Hecke K., Van Der Voort P., Krishnaraj C. (2024). Controlled Synthesis of Crystalline Metalated Porphyrin Covalent Organic Frameworks through an Assembler Approach. Chem. Mater..

[45] Wang Z., Zhou Z., Wang D., Deng C., Li J., Lin W. (2026). Bifunctional Covalent Organic Frameworks Enable Ambient Photocatalytic Oxidation via Synergistic Ligand-to-Metal Charge Transfer and O_2_ Activation. J. Am. Chem. Soc..

[46] Huang L., Liu Q., Wu W., Gao G., Zheng X., Wang J., Dong S. (2023). Identifying the active sites in unequal iron-nitrogen single-atom catalysts. Nat. Commun..

[47] Ren T., Lu K., Tao F., Ren H., Yan N., Miao J., Huang X., Zhang X. (2025). Phosphorus-induced single-atom iron coordination symmetry disruption for superior catalytic ozonation. Nat. Commun..

[48] Miao F., Wang Y., Zhou H., Zhong S., Lin J., Xu W., Yue X., Ren W., Zhang H., Wang S. (2026). Axial oxygen coordination drives spin-regulated electron transfer in single-atom Fe catalysts for selective pollutant transformation. Nat. Commun..

[49] Chen J.-H., Li W.-T., Cai K.-Y., Tu H.-J., Long Z.-T., Akhtar S., Liu L.-D. (2025). Proton-coupled electron transfer controls peroxide activation initiated by a solid-water interface. Nat. Commun..

[50] Yu D., Xu L., Fu K., Liu X., Wang S., Wu M., Lu W., Lv C., Luo J. (2024). Electronic structure modulation of iron sites with fluorine coordination enables ultra-effective H_2_O_2_ activation. Nat. Commun..

[51] Zhao K.-M., Wu D.-X., Wu W.-K., Nie J.-B., Geng F.-S., Li G., Shi H.-Y., Huang S.-C., Huang H., Zhang J. (2025). Identifying high-spin hydroxyl-coordinated Fe^3+^N_4_ as the active centre for acidic oxygen reduction using molecular model catalysts. Nat. Catal..

[52] Li Y., Zhang D., Wang P., Qu J., Zhan S. (2024). Superoxide radicals mediated by high-spin Fe catalysis for organic wastewater treatment. Proc. Natl. Acad. Sci. USA.

[53] Ebner K., Clark A.H., Saveleva V.A., Smolentsev G., Chen J., Ni L., Li J., Zitolo A., Jaouen F., Kramm U.I. (2022). Time-Resolved Potential-Induced Changes in Fe/N/C-Catalysts Studied by In Situ Modulation Excitation X-Ray Absorption Spectroscopy. Adv. Energy Mater..

[54] Zhu G., Zhao S., Yu Y., Liu K., Fan X., Quan X., Liu Y. (2026). Axial Fluorine Coordinated Fe Single-Atom Catalyst for Boosting Electro-Fenton Treatment of Organic Pollutants. Adv. Funct. Mater..

[55] Chen Y., Li X., Jiang Z., Yang J., Wang Z., Zhu M. (2026). Ultralow H_2_O_2_-Triggered Electron Transfer over Single-Atom Iron Catalysts for Long-Term Recyclable Fenton-Like Reactions. Angew. Chem. Int. Ed..

[56] Jiang K., Back S., Akey A.J., Xia C., Hu Y., Liang W., Schaak D., Stavitski E., Nørskov J.K., Siahrostami S. (2019). Highly selective oxygen reduction to hydrogen peroxide on transition metal single-atom coordination. Nat. Commun..

[57] Chen J.-H., Long Z.-T., Cai K.-Y., Tu H.-J., Wu H.-C., Bian F.-Q., Liu L.-D. (2026). Proton-Coupled Electron Transfer Governs Heterogeneous Fenton-like Reactivity, Modulation of Surface O-H Bond Dissociation Free Energy and Proton Activity. J. Am. Chem. Soc..

[58] Wang X. (2026). Proximity Engineering of Fe–N_4_ Twins for Oriented Generation of Singlet Oxygen for Hospital Wastewater Treatment. Angew. Chem. Int. Ed..

[59] Li H., Zhang X., Yang S., Sun Y., Qian J. (2024). Discerning the Relevance of Singlet Oxygen in Pollutant Degradation in Peroxymonosulfate Activation Processes. Environ. Sci. Technol..

[60] Meng Y., Liu Y.-Q., Wang C., Si Y., Wang Y.-J., Xia W.-Q., Liu T., Cao X., Guo Z.-Y., Chen J.-J. (2024). Nanoconfinement steers nonradical pathway transition in single atom fenton-like catalysis for improving oxidant utilization. Nat. Commun..

[61] Jiang X., Zhou B., Yang W., Chen J., Miao C., Guo Z., Li H., Hou Y., Xu X., Zhu L. (2024). Precise coordination of high-loading Fe single atoms with sulfur boosts selective generation of nonradicals. Proc. Natl. Acad. Sci. USA.

[62] Ren S., Wang Y., Shi L., Xu X., Zhong S., Hu K., Zhou H., Zhu Z.-S., Zhou P., Tian W. (2025). Transforming Plastics to Single Atom Catalysts for Peroxymonosulfate Activation, Axial Chloride Coordination Intensified Electron Transfer Pathway. Adv. Mater..

[63] Scalfi L., Becker M.R., Netz R.R., Bocquet M.-L. (2023). Enhanced interfacial water dissociation on a hydrated iron porphyrin single-atom catalyst in graphene. Commun. Chem..

[64] Bae G., Kim M.M., Han M.H., Cho J., Kim D.H., Sougrati M.-T., Kim J., Lee K.-S., Joo S.H., Goddard W.A. (2023). Unravelling the complex causality behind Fe-N-C degradation in fuel cells. Nat. Catal..

[65] Liu C., Li J., He X., Yue J., Chen M., Chen J.P. (2024). The “4 + 1” strategy fabrication of iron single-atom catalysts with selective high-valent iron-oxo species generation. Proc. Natl. Acad. Sci. USA.

[66] Zhang L., Cheng K., Yang Z., Zhang Y., Kubuki S., Bingham P.A., Yong Y.-C., Zhang B., Duan X. (2025). Deciphering the Origin of Higher Shell Coordination on Single Iron Catalysts for Resilient Modulating Persulfate Oxidation Into Singlet Oxygen Pathway. Adv. Funct. Mater..

[67] Wan W., Zhao Y., Wei S., Triana C.A., Li J., Arcifa A., Allen C.S., Cao R., Patzke G.R. (2021). Mechanistic insight into the active centers of single/dual-atom Ni/Fe-based oxygen electrocatalysts. Nat. Commun..

[68] Liu T., Xiao S., Li N., Chen J., Zhou X., Qian Y., Huang C.-H., Zhang Y. (2023). Water decontamination via nonradical process by nanoconfined Fenton-like catalysts. Nat. Commun..

[69] Zhang Y.-J., Huang G.-X., Winter L.R., Chen J.-J., Tian L., Mei S.-C., Zhang Z., Chen F., Guo Z.-Y., Ji R. (2022). Simultaneous nanocatalytic surface activation of pollutants and oxidants for highly efficient water decontamination. Nat. Commun..

[70] Qian Y., Li D., Han Y., Jiang H.-L. (2020). Photocatalytic Molecular Oxygen Activation by Regulating Excitonic Effects in Covalent Organic Frameworks. J. Am. Chem. Soc..

[71] Shi L.-J., Wang Z.-H., Zhang Y.-J., Chen J.-J., Li W.-W., Huang G.-X., Yu H.-Q. (2025). Anions-Impacted Water Purification from a Dual-Substrate Perspective. Environ. Sci. Technol..

[72] Yang X., Rosario-Ortiz F.L., Lei Y., Pan Y., Lei X., Westerhoff P. (2022). Multiple Roles of Dissolved Organic Matter in Advanced Oxidation Processes. Environ. Sci. Technol..

[73] Xie X., Li B., Xu P., Sougrati M.T., Garcia-Serres R., Cullen D.A., Kropf A.J., Xia F., Song M., Saha S. (2025). Unravelling the Stability Stressors of Atomically Dispersed Fe-N-C Oxygen Reduction Catalysts. J. Am. Chem. Soc..

[74] Hou Y., Zhou P., Liu F., Tong K., Lu Y., Li Z., Liang J., Tong M. (2024). Rigid covalent organic frameworks with thiazole linkage to boost oxygen activation for photocatalytic water purification. Nat. Commun..

[75] Duan P., Liu J., Chen L., Li M., Pan J., Zhang Z., Bai C., Chen X., Yu H., Chen F. (2025). Polymeric products deactivate carbon-based catalysts in catalytic oxidation reactions. Nat. Water..

